# Impact of a Novel, Low-Cost and Sustainable Health Education Program on the Knowledge, Attitudes, and Practices Related to Intestinal Schistosomiasis in School Children in a Hard-to-Reach District of Madagascar

**DOI:** 10.4269/ajtmh.21-0220

**Published:** 2022-01-10

**Authors:** Stephen A. Spencer, Emmanuel H. Andriamasy, Cortland Linder, James M. StJ. Penney, Jemima Henstridge-Blows, Hannah J. Russell, Kate Hyde, Caitlin Sheehy, Isla L. Young, Benedicte Sjoflot, Daniel A. L. Rakotomampianina, Anjara M. Nandimbiniaina, Gina U. Raderalazasoa, Tahiry N. Ranaivoson, Antsa Andrianiaina, Rasolofomanana S. M. Michèle, Zafera A. Rohe, Amaya L. Bustinduy, J. Russell Stothard, Sheena M. Cruickshank, Glenn T. Edosoa, Alain M. Rahetilahy

**Affiliations:** ^1^ North Bristol NHS Trust, Bristol, United Kingdom;; ^2^ The University of Manchester Faculty of Biology Medicine and Health, Manchester Academic Health Centre, Manchester, United Kingdom;; ^3^ Faculté de Médecine, Université d’Antananarivo, Antananarivo, Madagascar;; ^4^ Department of Clinical Research, London School of Hygiene and Tropical Medicine, London, United Kingdom;; ^5^ Department of Tropical Disease Biology, Liverpool School of Tropical Medicine, Liverpool, United Kingdom;; ^6^ World Health Organization, Madagascar Country Office, Antananarivo, Madagascar;; ^7^ Ministère de la Santé Publique de Madagascar, Antananarivo, Madagascar

## Abstract

Schistosomiasis control requires multisectoral approaches including praziquantel treatment, access to safe water, sanitation and hygiene, and health education. Community input can help ensure health education programs are culturally appropriate to effectively direct protective behavior change. This study reports on the three-stage development of an education program for Malagasy children, with an impact evaluation on their knowledge, attitudes, and practices (KAP) related to intestinal schistosomiasis. A cross-sectional study took place in 2017 with follow-up in 2018 in the hard-to-reach Marolambo district, Madagascar. A novel schistosomiasis education program (SEP) was designed in collaboration with researchers, stakeholders, and local community and included cartoon books, games, songs, puzzles, and blackboard lessons, costing $10 USD per school. KAP questionnaires were completed by 286 children pre-SEP and 273 children post-SEP in 2017, and by 385 and 337 children pre-SEP and post-SEP, respectively, in 2018. Improvements were observed in responses to all questions between pre- and post-education answers in 2017 (53–77%, *P* < 0.0001) and 2018 (72–98%, *P* < 0.0001) and in the pre-education answers between years (53–72%, *P* < 0.0001). Praziquantel mass drug administration attendance improved, rising from 64% to 91% (*P* < 0.0001), alongside improved latrine use, from 89% to 96% (*P* = 0.005). This community-consulted and -engaged SEP resulted in substantial improvements in children’s understanding of schistosomiasis, with improvements in praziquantel uptake and latrine use. Socioculturally tailored education programs can help gain schistosomiasis control. Continued investment in SEP will help promote the future well-being of children through increased participation in control and treatment activities.

## INTRODUCTION

Effective health education is a vital component of any integrated public health campaign due to its role in driving health-related behavior changes.^1^ However, many remote communities where neglected tropical diseases (NTDs) occur do not have access to sufficient health infrastructure, resources, or health education.^2^^,^^3^ The World Health Assembly resolution WHA54.19 urged member states to promote access to health education through intersectoral collaboration, a message replicated in WHO guidelines.^4^^–^^6^ Although externally driven initiatives in community health education can have a positive impact, this is rarely sustained beyond termination of funding.^7^ Generic public health approaches often do not cater to local cultural practices and are less likely to succeed than those that do. Active community encouragement and participation, with facilitation, is necessary for better tailoring of sustainable approaches to targeted communities.^8^^,^^9^

Schistosomiasis is a NTD that affects approximately 290 million people worldwide and results in 1.4 million disability-adjusted life years lost each year.^10^^,^^11^ *Schistosoma mansoni* infection causes intestinal schistosomiasis, which can lead to anemia, stunting, wasting, reduced exercise performance, and potentially fatal conditions such as liver fibrosis.^12^ Humans are infected with trematode flukes through contact with fresh water infested with infectious larvae (cercariae), which mature in the human host and breed to produce eggs. Transmission results from an infected person defecating *S. mansoni* eggs back into water sources. Here, miracidia are released from the eggs and infect certain freshwater snails. The obligatory intermediate snail hosts in turn release numerous cercariae into fresh water, where they can come into contact with humans.^13^ The treatment, praziquantel (PZQ), is delivered as annual mass drug administration (MDA) to high prevalence (> 50%) locations as preventive chemotherapy.^5^

Optimal disease prevention, control, and acceptance of regular PZQ treatment relies on sustained and appropriate knowledge, attitude, and practices (KAP) in the community.^14^^,^^15^ For example, in Uganda, only 31% of people treated in 2004 for schistosomiasis were willing to return for treatment with PZQ at the MDA due to misconceptions about side effects of PZQ and the need for PZQ while being symptom free.^16^ Crucially, appropriate health education can mobilize and empower communities suffering from NTDs to identify or develop appropriate sustainable solutions in preventing disease and becoming more self-sufficient.^17^ Although there are reports of informal and formal schistosomiasis education through posters, radio, community gatherings, and games,^18^^,^^19^ in many endemic areas, there are no formal schistosomiasis education programs (SEPs) and no educational resources available for teachers or health workers, often due to insufficient finances for national NTD control programs.^20^^–^^23^ Among education initiatives that exist, some have failed, overlooking information on baseline KAP about disease or having inadequate focus on local sociocultural, ethnicity, language, and behavioral factors (or both), which risks nonengagement with prevention or control activities.^24^ Including community peers in the design and delivery of interventions is therefore fundamental to ensure processes take into account these local factors.

Marolambo is a highly remote and hard-to-reach rainforest district in Madagascar, hyperendemic for intestinal schistosomiasis, with an 88% to 98% prevalence of *S. mansoni* infection and high burden of disease among school-age children,^25^^–^^27^ and 67% prevalence among preschool-age children.^28^ MADagascar mEdical eXpeditions (MADEX) have been working with the communities in Marolambo since 2015 to reduce the burden of schistosomiasis and conduct locally appropriate implementation research. Through collaborations with researchers, community members, schools, and stakeholders, we have coproduced an SEP to improve understanding and promote protective behavior, specifically: 1) encourage compliance with treatment, 2) reduce contact with infested water, and 3) improve hygiene and use of latrines. Here we evaluate the impact of our newly designed SEP, using KAP questionnaires to assess for improvements in understanding and behavior relating to schistosomiasis transmission, prevention, and treatment.

## MATERIALS AND METHODS

### Study area and population.

This study was carried out in six villages in the Marolambo district: Marolambo, Ampasimbola, Ambohitelo, Marofatsy, Vohidamba, and Betampona. Marolambo is a remote district in the east of Madagascar (Supplemental Figure 1), with an estimated population of 215,000, although limited anthropometric data exist. The villages were chosen purposively based on the advice from local officials that the safety of the team could not be guaranteed in other villages in the region. In each study village selected, there is a single government school, overseen by district education officials in Marolambo village. In the Marolambo village, private schools exist in addition to the government school. Altogether, there are 290 schools across 190 villages in the district. There are no formal government-led education programs on schistosomiasis or MDA in Madagascar.

Malagasy and French are the official languages of Madagascar and are taught in schools, including in rural villages of Marolambo where a local dialect, Betsimisaraka Atsimo, is also commonly spoken. Communities in the Marolambo District rely on environmental water from the Nosivolo River for drinking, cooking, washing, and transport (Supplemental Figure 1). There are gravitational water supply systems that provide water through a series of pipes from streams to the villages. However, these are prone to environmental damage and are rarely repaired, meaning they are unreliable sources of water. Villages and schools have simple pit latrines, built from mud, sticks, or concrete (Supplemental Figure 2).

Following our research in 2015 showing a high prevalence of *S. mansoni* in the six villages, the Ministry of Health, Madagascar, has provided MDA to all school-age children in the district. Before this, there had not been an MDA in the Marolambo district since 2008.^25^

### Study design.

This schistosomiasis education study was a nested component of a research program on *S. mansoni* prevalence and infection intensity in response to MDA, using repeated annual cross-sectional studies in the same villages, the methodology of which has been previously published.^27^ For the current study, the first education-based cross-sectional study took place in June 2017 with a follow up cross-sectional study in June 2018. A specialized SEP was designed and delivered to all school-age children over 1 day in each village. A KAP questionnaire was designed to measure and quantify baseline schistosomiasis understanding and behavioral practices and the impact of the SEP. Each year between 50 and 80 children aged 5 to 14 were invited to complete the pre- and post-KAP questionnaire; this was the maximum possible within the logistical constraints of time and resources. Participants were selected randomly from an anonymized school register and stratified to give approximately equal numbers for age and gender. Before 2017, there had been no formal schistosomiasis education in the region.

### KAP questionnaires.

A pilot KAP questionnaire was designed with input from Malagasy medical students and local leaders in 2015. It was written in English (Supplemental File 1) and translated into Betsimisaraka Atsimo (the local Malagasy dialect) by three Malagasy doctors who reached a consensus with the translation. The pilot KAP was pretested among children aged 5 to 14 in Marolambo in 2016, and corrections to both the content and delivery in language were made using informal feedback from participants, interviewers, and teachers. An effort was made to minimize scientific terms and use easily understandable phrases so it could be understood by all targeted age groups; the only scientific word, “schistosomiasis,” was translated into the local vernacular “Bilharziose.”

The formal KAP questionnaires were completed by participants the day before and after delivery of the SEP in each village in June 2017 and June 2018. Questions were read from the questionnaire by the Malagasy researchers and verbal responses recorded in Malagasy, then back-translated into English. Participants were not time pressured, and questions were rephrased to facilitate understanding, particularly in young or illiterate participants. Each child was asked away from the presence of others to limit external influence and thereby reduce bias. Each answer was marked as correct or incorrect by a single investigator (to prevent interobserver variation) and checked by a second investigator to ensure agreement and prevent misclassification. If there was a discrepancy in marking, the original answer was reviewed again and a consensus reached. Participant answers relating to questions on attendance to the previous year’s MDA were verified by checking local MDA attendance registers.

To establish a control group, MDA register data were collected post hoc from six comparable schools in the Marolambo district that did not participate in this MADEX-SEP study: Lycée Marolambo, Center d’Enseignement Général Marolambo, École FLM 1, École FLM 2, CESAM, and École Papillion. MDA attendance from these nonparticipating (SEP-naïve) schools was compared with MDA attendance from SEP-participating schools to determine the impact of the SEP on MDA attendance.

### Three-stage development and delivery of the SEP.

**Figure 1.**
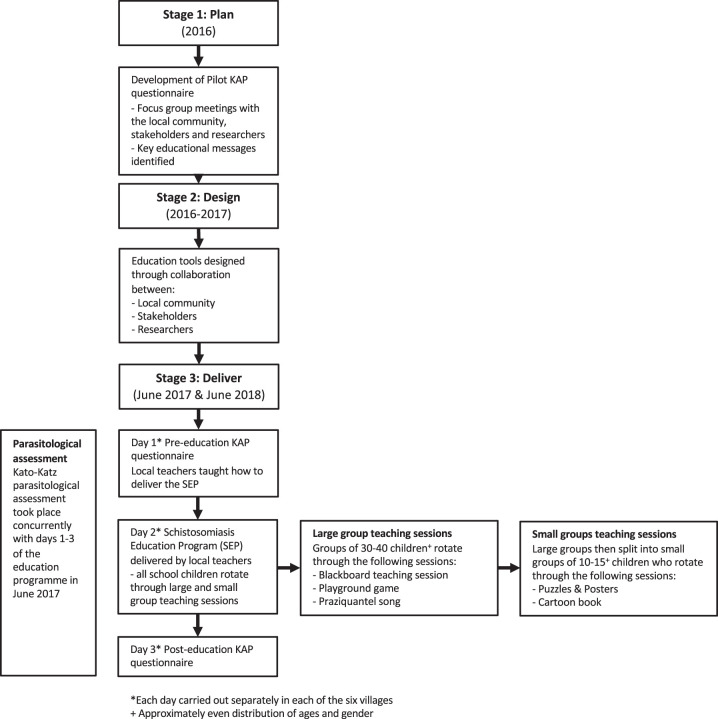
Three-stage development and delivery of the schistosomiasis education program (SEP). KAP = knowledge, attitudes, and practices.

The design, development, and delivery of the education program involved three stages (Figure 1) in partnership with the local community, teachers, researchers, and governmental authorities. This approach was informed by prior work on health education with immigrant communities,^29^ and the human-centered design model for public health interventions.^30^ The SEP was designed using the stages of change model.^31^ The SEP aimed to target the precontemplation stage where the individuals and audience are not aware of the need for change or improvement. Consciousness raising, dramatic relief, and environmental reevaluation are the processes addressed by the repeated expeditions and education implementation. The community members worked alongside the research team from start to finish to increase the approachability of the research team and work as an intermediary between researchers and the local community.^32^

#### Stage 1: Plan (2016).

Baseline understanding and prior perceptions about disease were ascertained from the 2016 pilot KAP survey and used to guide key messages of the SEP. After this, focus group discussions (FGDs) were held in 2016 in each of the six villages, which included representatives of the local communities: all local traditional and elected leaders attended as well as all school teachers and health workers. Community members, parents, and children were also invited to attend through convenience sampling after village announcements. Discussions in the FGDs established the importance of schistosomiasis education and highlighted that misconceptions of PZQ treatment jeopardized attendance to MDA.^33^^,^^34^ On the basis of the opinions of the FGDs and the UK and Malagasy research ethics committees, it was agreed that SEP should be offered to all children to maximize attendance at the MDA. Educational posters and classroom teaching were pretested on children aged 5 to 14 who attended the FGD. Key components of the education program were then discussed, including education tools, sustainability, durability, and reach. Educational messages were designed to cover prevention, transmission, and treatment. To invite all children in the community to the SEP, in the month before the MADEX-SEP study, weekly announcements were made to the community via megaphone and the made daily the week before the SEP.

#### Stage 2: Design (2016–2017).

The education program was designed through collaboration with the University of Manchester, Madagascar Ministry of Health, students and doctors from the University of Antananarivo, peer community members, local doctors, and teachers. A combination of didactic, interactive and kinesthetic learning methods were created so that the program catered to different ages and learning modalities. The education program used the following educational tools to teach on schistosomiasis transmission, prevention, and treatment:

1. Schistosomiasis transmission and prevention (with teaching on parasite lifecycle) using infographics, traditional education tools, and gameplay:
  A. Posters and puzzles (Supplemental Figures 3–5)
  B. Blackboard classroom teaching (Supplemental Figure 6)
  C. Cartoon book (*Scolly the Schisto Bug*; Supplemental Figure 7)
  D. Playground game (based on the lifecycle of the *S. mansoni* parasites).
2. Schistosomiasis treatment (and mass drug administration) using music:
  E. Schistosomiasis song

#### Stage 3: Delivery (June 2017 and June 2018).

In June 2017 and June 2018, teaching was delivered by both Malagasy members of the MADEX team and local teachers. First, local teachers were taught how to teach the SEP by MADEX doctors and scientists. Local teachers then delivered the SEP to all children in the local dialect, observed by Malagasy team members. All children rotated through each of the five educational activities. Because of the logistical demands of the research expedition in such a remote environment, the SEP delivery was limited to 1 day in each village. Head teachers were instructed how to train new teachers, and all teachers were encouraged to continue teaching the SEP when appropriate thereafter.

The overall cost for each pack was $10 (USD) per village, $7.50 for materials and $2.50 for printing and binding. All resources were left in schools of each village to allow for future use and continuity, including the lyrics for the songs to allow them to be easily relearned if forgotten.

### Parasitological examination.

Parasitological examinations were performed in June 2017 (before the annual MDA) to determine the number of study participants infected with *S. mansoni*. Participants were provided with a single sterile stool container to provide a single fecal sample. Two thick smears (containing 41.7 mg of stool) were prepared from each stool sample using the Kato-Katz (KK; Vestergaard-Frandsen, Lausanne, Switzerland) method.^35^ Homogenization of faecal samples was carried out before slides were prepared to increase accurate detection of *S. mansoni* eggs.^36^ Each slide was examined under light microscopy by researchers with prior training and experience in KK microscopy to count *S. mansoni* eggs. To ensure reliability one in every 10 slides was reexamined by a second researcher who was blinded to the findings from the first reading. If a difference was identified, the slide was reexamined and a consensus reached. *S. haematobium* was not tested for because a previous study demonstrated that this species is not endemic in the Marolambo district.^25^ There was no parasitological assessment in 2018 because the district received an MDA in the weeks leading up to the research expedition.

### Ethical approval.

Ethical approval was granted by the University of Manchester Research Ethics Committee (UREC3 #16153) and the Ministère de la Santé Publique de Madagascar (No. 621/001/2016-CEER/INSPC). Written consent for participation in the study was obtained from the child and their parent/guardian. All data were anonymized.

### Statistical analysis.

Questionnaires were completed on paper, and answers were transferred to Microsoft Excel (Redmond, WA). Statistical analyses were performed using StataCorp 2017 (Stata Statistical Software 15; StataCorp LLC, College Station, TX). chi-square analyses were used to 1) examine infection prevalence between groups of children when stratified by river contact behavior, 2) assess for differences in the percentage of correct responses in questionnaire data between years, and 3) compare MDA attendance among children from the six MADEX SEP-participating schools to children from six nonparticipating SEP-naïve schools. Matched McNemar’s chi-square analyses were performed on paired within-year questionnaire data (comparing pre- versus posteducation KAP responses). Kolmogorov-Smirnov tests were used to test for differences in male and female answers and Spearman’s rank to assess for correlation between age and percentage of correct responses.

## RESULTS

There were 286 of 300 (95%) respondents for the preeducation questionnaire in 2017 of whom 273 of 286 (95%) returned to complete the posteducation questionnaire. In 2018, 385 of 400 (96%) children attended the preeducation questionnaire, and 337 of 385 (88%) completed the post-questionnaire (Table 1).

**Table 1:** Number of study participants recruited each year, by age, gender, and location

	2017				2018			
	Preeducation		Posteducation		Preeducation		Posteducation	
	*n* (%)	Male, *n* (%)	*n* (%)	Male, *n* (%)	*n* (%)	Male, *n* (%)	*n* (%)	Male, *n* (%)
Overall response rate	286/300 (95%)	145/286 (51%)	273/286 (95%)	139/273 (51%)	385/400 (96%)	167/385 (43%)	337/385 (88%)	143/337 (42%)
Age								
5	24 (8%)	16	23 (8%)	16	39 (10%)	19	31 (9%)	13
6	26 (9%)	12	25 (9%)	12	38 (10%)	19	35 (10%)	17
7	28 (10%)	15	27 (10%)	14	37 (10%)	17	35 (10%)	16
8	29 (10%)	13	26 (10%)	11	37 (10%)	14	34 (10%)	13
9	31 (11%)	16	31 (11%)	16	37 (10%)	18	35 (10%)	16
10	30 (11%)	15	29 (11%)	14	38 (10%)	17	33 (10%)	15
11	28 (10%)	14	27 (10%)	14	42 (11%)	17	37 (11%)	14
12	30 (11%)	14	29 (11%)	14	36 (9%)	14	32 (10%)	14
13	30 (11%)	14	28 (10%)	14	38 (10%)	13	31 (9%)	11
14	30 (11%)	16	28 (10%)	14	43 (11%)	19	34 (10%)	14
Location								
Marolambo	50 (18%)	25	45 (17%)	23	58 (15%)	25	51 (15%)	23
Ampasimbola	48 (17%)	23	45 (17%)	21	59 (15%)	13	55 (16%)	12
Ambohitelo	44 (15%)	24	44 (16%)	24	60 (16%)	29	56 (17%)	28
Marofatsy	45 (16%)	22	45 (17%)	22	65 (17%)	27	58 (17%)	26
Vohidamba	49 (17%)	25	47 (17%)	24	78 (20%)	41	65 (19%)	32
Betampona	50 (18%)	26	47 (17%)	25	65 (17%)	32	52 (15%)	32

### Knowledge and attitudes.

The knowledge- and attitudes-related questions revealed variable and mixed answers in the preeducation questionnaire in 2017; the percentage of correct answers ranged from 11% and 79% (Table 2). Improvements were seen in responses to all questions between the pre- and posteducation answers in both 2017 (53–77%, *P* < 0.0001, odds ratio [OR]: 3.05, 95% confidence interval [CI]: 2.68–3.46) and 2018 (72–98%, *P* < 0.0001, OR: 15.00, 95% CI: 12.05–18.84). The percentage of correct preeducation answers also improved between 2017 and 2018 from 53% to 72% (*P* < 0.0001, OR: 2.32, 95% CI: 2.11–2.56).

**Table 2:** Percentage of correct responses to pre- and posteducation questionnaire knowledge and attitude components, by year

	2017				2018				2017 vs. 2018	
	Pre	Post	Pre vs. post^*^		Pre	Post	Pre vs. post^*^		Pre 2017 vs. pre 2018	
	*n* of 286 (%)	*n* of 273 (%)	*P*	OR (95% CI)	*n* of 385	*n* of 337	*P*	OR (95% CI)	*P*	OR (95% CI)
Knowledge										
Have you heard of schistosomiasis before?	226 (79%)				349 (91%)	337 (100%)	<0.0001	. (8.46)	<0.0001	2.57 (1.61–4.14)
What is schistosomiasis?	154 (54%)				312 (81%)	336 (100%)	<0.0001	. (16.58)	<0.0001	3.66 (2.56–5.25)
How do you think you get infected?	149 (52%)	227 (83%)	<0.0001	9.60 (5.00–20.66)	290 (75%)	331 (98%)	<0.0001	. (20.65)	<0.0001	2.80 (1.99–3.95)
What are the symptoms of schistosomiasis?	185 (65%)	227 (83%)	<0.0001	4.47 (2.52–8.42)	306 (79%)	331 (98%)	<0.0001	70.00 (12.16–280.39)	<0.0001	2.11 (1.47–3.04)
How long are you sick for?	32 (11%)	152 (56%)	<0.0001	18.14 (8.55–46.04)	110 (29%)	323 (96%)	<0.0001	230.00 (40.87–912.35)	<0.0001	3.18 (2.04–5.04)
Can you avoid getting infected with schistosomiasis?	151 (53%)	239 (88%)	<0.0001	12.88 (6.29–30.62)	293 (76%)	330 (98%)	<0.0001	38.00 (10.16–319.46)	<0.0001	2.85 (2.02–4.02)
How do you prevent getting infected with schistosomiasis?	92 (32%)	216 (79%)	<0.0001	19.42 (9.18–49.24)	241 (63%)	324 (96%)	<0.0001	39.67 (13.24–195.08)	<0.0001	3.53 (2.52–4.94)
Is there treatment?	225 (79%)	257 (94%	<0.0001	7.67 (3.26–21.97)	321 (83%)	332 (99%)	<0.0001	24.50 (6.43–207.99)	0.121	1.36 (0.90–2.05)
What is the treatment?	52 (18%)	224 (82%)	<0.0001	. (47.21)	191 (50%)	318 (94%)	<0.0001	74.00 (20.13–616.73)	<0.0001	4.43 (3.05–6.48)
Attitude										
Do you worry about getting schistosomiasis?	209 (73%)				322 (84%)	323 (96%)	<0.0001	45.00 (7.67–181.64)	0.001	1.88 (1.27–2.79)
Do you think schistosomiasis is serious?	194 (68%)				330 (86%)	330 (98%)	<0.0001	11 (4.35–35.57)	<0.0001	2.85 (1.92–4.24)
Total	1,669/3,149 (53%)	1,542/1,991 (77%)	<0.0001	3.05 (2.68–3.46)	3,065/4,235 (72%)	3,615/3,707 (98%)	<0.0001	15.00 (12.05–18.84)	<0.0001	2.32 (2.11–2.56)

CI = confidence interval; OR = odds ratio.

* Results from paired McNemar’s χ^2^ data analyse

Older children were more likely to provide correct answers (2017 preeducation: *r* = 0.45, *P* < 0.0001; 2017 posteducation: *r* = 0.49, *P* = 0.0001; 2018 preeducation: *r* = 0.51, *P* < 0.001; 2018 posteducation: *r* = 0.30, *P* < 0.0001).

There were no differences in scores between male and female participants (2017: *P* = 0.98; 2018: *P* = 0.97).

### Practices.

**Figure 2.**
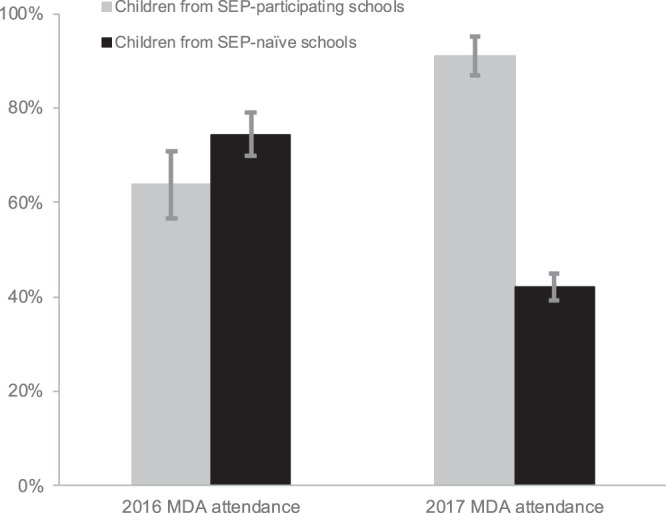
Changes in attendance to mass drug administration (MDA) by year. Bars represent the percent of children who attended each MDA, comparing study-enrolled children from schistosomiasis education program (SEP)-participating schools (*N* = 286 for MDA in 2016, *N* = 385 for MDA in 2017) to children from SEP-naïve schools (*N* = 1,185 for MDA in 2016, *N* = 1179 for MDA in 2017). Error bars represent 95% confidence intervals. Chi-square analyses demonstrated no difference in the MDA attendance before the SEP in 2016 (*P* = 0.153, odds ratio [OR]: 0.86, 95% confidence interval [CI]: 0.69–1.06) and after the SEP in 2017, higher MDA attendance among children from SEP-participating schools than children from SEP-naïve schools (*P* < 0.0001, OR: 2.17, 95% CI: 1.79–2.62).

Questions relating to practices—namely, MDA attendance, river contact behavior, and place of defecation—were only asked in the preeducation questionnaires each year. Among the SEP-participating children, there was an increase in MDA attendance from 64% (pre-SEP; 2016 MDA) to 91% (post-SEP; 2017 MDA; *P* < 0.0001; OR: 5.78 95% CI: 3.60–9.29; Figure 2). Despite community announcements and invitation to each SEP, only pupils from the MADEX SEP-participating schools attended each SEP. MDA attendance data were also collected from six SEP-naïve schools from both 2016 and 2017, totaling 1,185 children in 2016, and 1,179 children in 2017. In 2016, MDA attendance did not differ between children from SEP-participating schools and SEP-naïve schools (64% versus 74%, respectively; *P* = 0.153, OR: 0.86 95% CI: 0.69–1.06). After the delivery of the SEP in 2017, the 91% attendance from SEP-participating schools was significantly higher than the 42% attendance rate among children from SEP-naïve schools (*P* < 0.0001, OR: 2.17 95% CI: 1.79–2.62). Among SEP-naïve schools, the decline in MDA attendance from 74% to 42% was significant (*P* < 0.0001, OR: 0.25 95% CI: 0.21–0.30).

**Figure 3.**
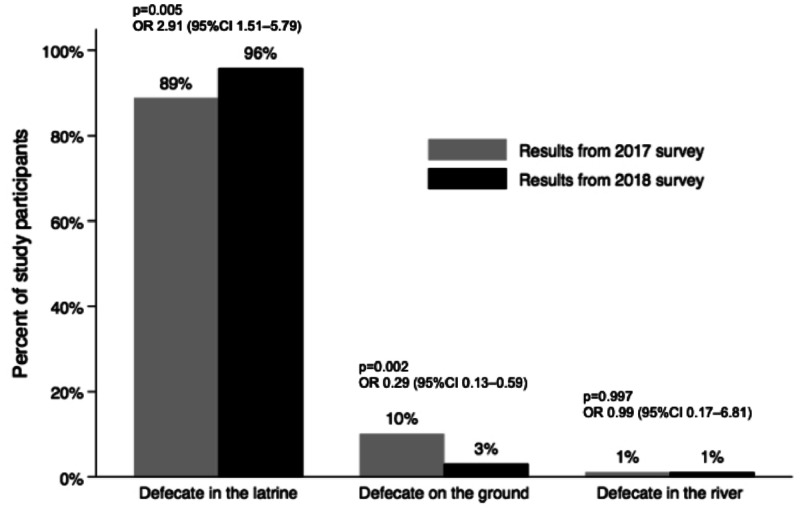
Changes in self-reported place of defecation by year. A questionnaire was used to ask children whether they defecated in latrines, on the ground, or in the river. Results from the survey in 2017 reflect pre- schistosomiasis education program (SEP) behaviors and results from the 2018 survey reflect behaviors after the 2017 SEP and before the 2018 SEP. Outputs from chi-square analyses are shown.

The percentage of respondents defecating in latrines improved from 89% in 2017 to 96% in 2018 (*P* = 0.005), and the percentage reporting to defecating on the ground reduced from 10% to 3% (*P* = 0.002; Figure 3). In contrast, the percentage of participants who declared having had daily contact with the Nosivolo River increased from 40% to 80% (*P* < 0.001), and the percentage with no contact with the river decreased from 24% to 5% (*P* < 0.001; Table 3).

**Table 3:** Changes in frequency of river contact between 2017 and 2018 and association between river contact frequency and prevalence of *Schistosoma mansoni* infection from 2017

Frequency of going to the river	2017 *n* (%)	2018 *n* (%)	*P*	OR (95% CI)	*% S. m.+* 2017^*^	*P*	OR (95% CI)
Never	69 (24%)	20 (5%)	< 0.001	0.17 (0.10–0.30)	61%	Ref	Ref
Less than weekly	33 (12%)	35 (9%)	0.30	0.77 (0.45–1.31)	82%	0.04	2.85 (1.04–7.85)
Multiple times/week	70 (24%)	21 (5%)	< 0.001	0.18 (0.10–0.30)	87%	0.001	4.22 (1.80–9.95)
Daily or more	114 (40%)	308 (80%)	< 0.001	6.11 (4.27–8.77)	82%	0.003	2.89 (1.45–5.75)

CI = confidence interval; OR = odds ratio; Ref = reference.

* *S. m.*+ = positive *S. mansoni* infection (diagnosed by Kato-Katz microscopy)

### Parasitology.

In 2017 there was evidence of egg-patent infection in 228 or 293 (78%; 95% CI: 73–82%) of children. Among children who had no contact with the Nosivolo River, the prevalence of egg-patent infection was 61% (Table 3); this was significantly lower than those who did have contact with the Nosivolo River, either less than weekly (82%; OR: 2.85, 95% CI: 1.04–7.85), more than weekly (87%; OR: 4.22, 95% CI: 1.80–9.95) or daily (82%; OR: 2.89, 95% CI: 1.45–5.75).

## DISCUSSION

This is the first study to attempt and successfully implement a three-stage design with community consultation, engagement, and participation, to deliver a socioculturally appropriate SEP in a hard-to-reach area. In addition, this is the first assessment of KAP relating to intestinal schistosomiasis in a hyperendemic area of Madagascar. Our initial assessments in 2017 demonstrated large gaps in knowledge of schistosomiasis, similar to reports elsewhere.^22^ After the SEP, improvements were observed in understanding of schistosomiasis, MDA attendance, and latrine use.

Children from SEP-participating schools had 91% MDA attendance after the SEP—more than double the attendance rates compared with children from SEP-naïve schools. Among SEP-naive schools, MDA attendance fell from 74% (2016) to 42% (2017), which likely reflects misconceptions regarding PZQ, fears of side effects, and lack of perceived benefits in the absence of education, as ascertained during the focus group discussions and reported in studies elsewhere in Africa.^16^^,^^24^^,^^33^^,^^34^ In our study, only school-attending children participated in the SEP, despite attempts to include non–school-attending children. In hyperendemic settings, strategies are needed to effectively disseminate information and maximize participation in MDA, particularly among non–school-attending children who often have higher disease burdens.^37^^,^^38^

Our data highlight that frequent water contact is concerning, showing that children who had contact with the river had a higher likelihood of infection with *S. mansoni* than those with no river contact. Our work with this community highlights the dependence on the river and supports renewed community-directed efforts to ensure access to clean water in this area. Despite our educational efforts, there was an increase in reported contact with infested water between 2017 and 2018. This observation does not negate the use of our education program and may simply reflect changing infrastructure. For example, the number of gravitational water sources in the six villages fell from 27 to nine between 2017 and 2018 as a result of environmental damage and the lack of resources available for repairs to be made. Similar studies in Egypt, Zimbabwe, and Ethiopia showed that improved knowledge did not always result in reduced water contact, particularly where safe water supplies were not available.^24^ In Brazil, despite good understanding of intestinal schistosomiasis, 76.2% of children reported contact with infested water, and the investigators reported increased odds of egg-patient infection by 1.75 in these children, comparable to findings from our study.^39^ Importantly, the Marolambo district gravitational water sources are not filtered or treated and therefore may contain cercariae if they originate from contaminated water sources (Supplemental Figure 2). Additionally, adults and children in the Marolambo district have regular contact with the Nosivolo River for work (panning for gold), as well as with rice paddies for farming; an inspection in 2019 found *S. mansoni* cercariae in both rice paddies and freshwater sources.^27^^,^^40^ The adult population are therefore likely to contribute to local transmission through these water contact activities. Although increased latrine use was noted, many of the pit latrines in the villages are close to the Nosivolo River (Supplemental Figure 2), which may result in direct contamination. Improving sanitation alongside health education may help reduce schistosomiasis transmission.^41^

This SEP consisted of five educational tools, including didactic and interactive school-based blackboard lessons, group activities solving puzzles and jigsaw, songs, games, and cartoon books. Previous research has demonstrated improvements in schistosomiasis knowledge after a similar range of educational activities.^19^^,^^42^^–^^45^ For example, in Côte d’Ivoire, a cartoon book produced in collaboration with local children and teachers was well received and resulted in improvements in knowledge of helminthiasis and diarrheal diseases.^45^ Comparable educational activities in Zanzibar resulted in improved MDA attendance and reduced contact with infested water.^46^ Our study did not assess whether one particular teaching method was more effective than another. Furthermore, this educational toolkit was delivered to children aged 5 to 14; future work could compare the efficacy and age-appropriateness of specific educational tools.

Although there was no control comparator for knowledge assessments, we believe improvements in schistosomiasis understanding are likely to be related to our teaching program because there were no other health education programs in this area before or during the study. Our KAP questionnaire may have be subject to responder or observer bias, although we attempted to limit this in our methodology. The cross-sectional study design assesses the impact of the SEP intervention on school populations. Although there may have been variability in the teaching standards between schools, all teaching was observed to minimize this. Despite these limitations, this health engagement study puts forward the first data on a successful community participation in a context-appropriate health education program in rural Madagascar, with promising findings that can help benchmark future health education delivery in rural areas and offer feasible inclusion in the school curriculum in endemic regions.

Previous studies have shown how local involvement in health education helps interrupt transmission of schistosomiasis.^14^^,^^17^^,^^47^ In line with this, our SEP was developed with ongoing engagement and feedback from the community, which was fundamental to create a culturally appropriate and effective health education program.^48^ We used FGDs to help define the sociocultural context and identify barriers and opportunities to schistosomiasis-control programs.^49^ Involving teachers in this process guided lesson design and delivery. FGDs with teachers have been similarly used in Tanzania to develop song, drama, and play into lessons and improve understanding of schistosomiasis.^43^ These steps are especially important in populations living in particularly hard-to-reach or remote areas such as Marolambo, where a generic approach is unlikely to succeed.

The SEP was delivered by community peer members, who can help with approachability, acceptability, and reach of both health education and research projects.^32^^,^^50^ Furthermore, involving communities can help generate ownership of the project, which is vital to improve long-term outcomes and shift away from reliance on external organisations.^30^^,^^51^ In Brazil, a SEP that empowered teachers, students, and families to take more ownership over their own health promoted a more sustainable control of schistosomiasis within the community.^52^ In Zanzibar, local religious leaders have also helped to drive behavioral change related to schistosomiasis.^53^

The SEP was designed to be low-cost, durable, sustainable, and easily maintained by the community. Educational tools such as the song and the game were zero cost and can continue to be played by the children after the SEP. School teachers were also trained in delivery of the education program. This allows for the repetition of key educational messages over time and helps to retain knowledge. Enhanced awareness of infection among children may further improve knowledge in the wider community due to dialogue between children and their families, leading to wider changes in the area. Previous work on education awareness has shown that immigrant communities have been empowered to share newly learned knowledge with their families, that teachers often feel enabled to continue sharing new knowledge independently,^29^ and that this legacy is sustained (S. Cruickshank, personal communication). Future studies in Madagascar could assess the impact of this set of resources on the teachers and communities. The cost of materials was $10 USD per pack (one pack per school, one school per village). This does not, however, include salaries for teachers or transport costs of materials; a lack of dedicated time and funding for regular schistosomiasis education was recognized as a significant barrier to ongoing teaching throughout the year. Although materials were designed to last several years without needing to be replaced, future investment into SEPs is vital for both scale-up and long-term sustainability.

Successful scale-up of education activities will require attention to the culture, ethnicity, and language of the target population. This is likely to be a particular challenge in countries comprising multiple ethnic and cultural groups, such as Madagascar. The three-stepped, community-engaged methodological approach discussed in this study provides a framework that can be followed to adapt the SEP to allow for scale-up to other communities and can also enable expansion to tackle the information and education of other neglected tropical diseases.

## CONCLUSION

This health education strategy resulted in a positive impact on understanding of intestinal schistosomiasis among schoolchildren. Importantly, MDA attendance improved among children who attended the SEP in contrast to a fall in MDA attendance among SEP-naïve children in the same district. Continued investment in SEP is required to promote future well-being of children through increased participation in disease control and treatment activities. It is also vital for schistosomiasis control programs to ensure that there is sufficient access to safe water, sanitation, and hygiene, in addition to the necessary health education, treatment, and snail control in high-transmission, hyperendemic zones such as this.

## Supplemental Material


Supplemental materials
