# Supplementary material for: Impact of a Novel, Low-Cost and Sustainable Health Education Program on the Knowledge, Attitudes, and Practices Related to Intestinal Schistosomiasis in School Children in a Hard-to-Reach District of Madagascar
Source: Am J Trop Med Hyg. 2022 Jan 10;106(2):685–94. doi: 10.4269/ajtmh.21-0220 (PMC8832942; doi:10.4269/ajtmh.21-0220)
Supplement: Supplementary file 1 [file tpmd210220.SD1.pdf]

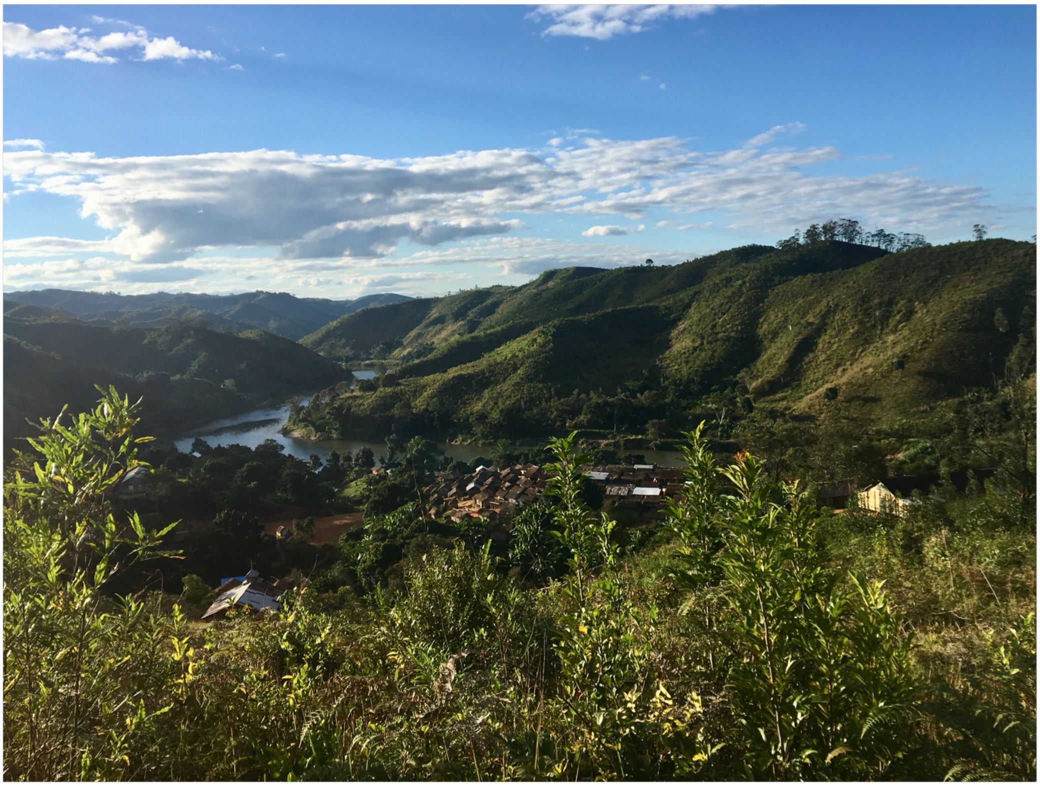

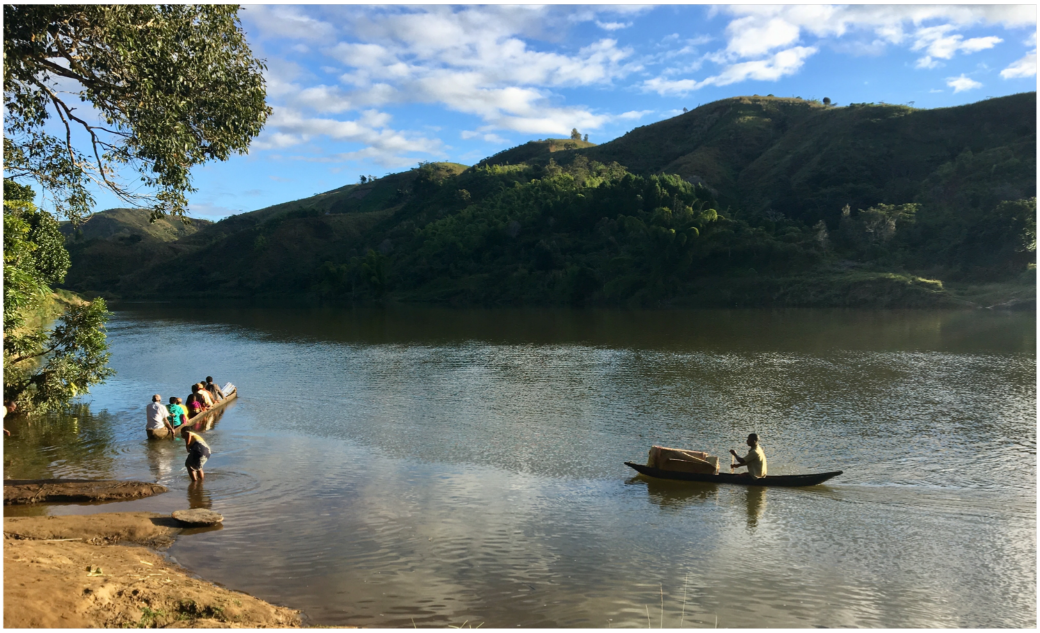

Fig S1. Photographs from Marolambo. Top: the remote village, Betampona in the Marolambo district. Bottom: River crossing in Marofatsy, Marolambo, showing transport canoes and water contact.

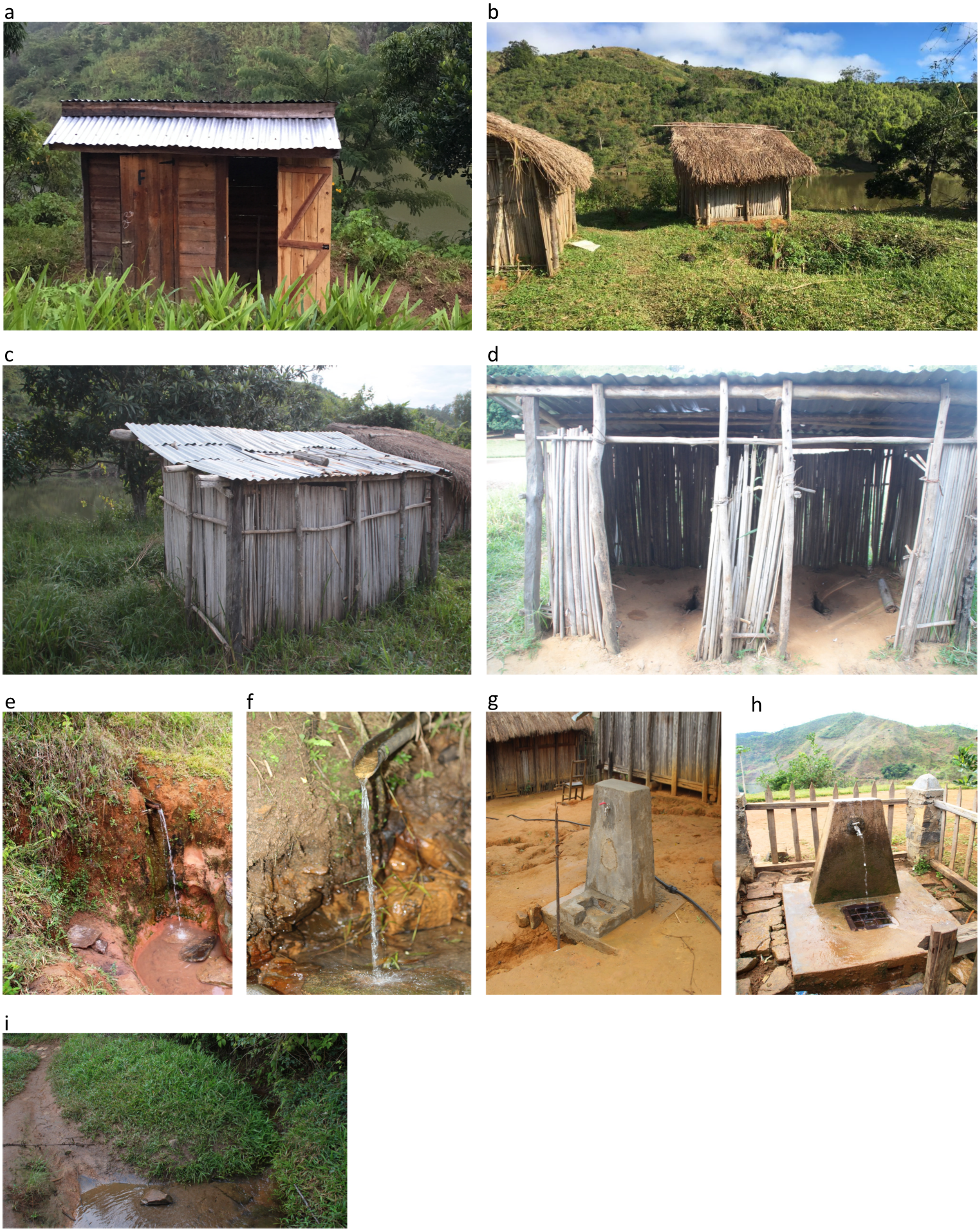

Fig S2. WASH Photos. Photos of pit latrines (photos a - d), gravitational water supplies (e - h), and stream used as a water source (i) taken from villages in the Marolambo district, Madagascar. a - pit latrine in Ambohitelo; b, c & d – pit latrines in Marofatsy; e - gravitational water supply in Vohidamba; f – close up of gravitational water supply in Ampasimbola; g – gravitational water supply with tap in Betampona; h – gravitational water supply with tap in Vohidamba; i - stream used as a source of water in Ambohitelo. Photos taken by Stephen Spencer (a & f; June 2017), James Penney (c-e, g-i; June 2017) and Heather Lawson (b; June 2019).

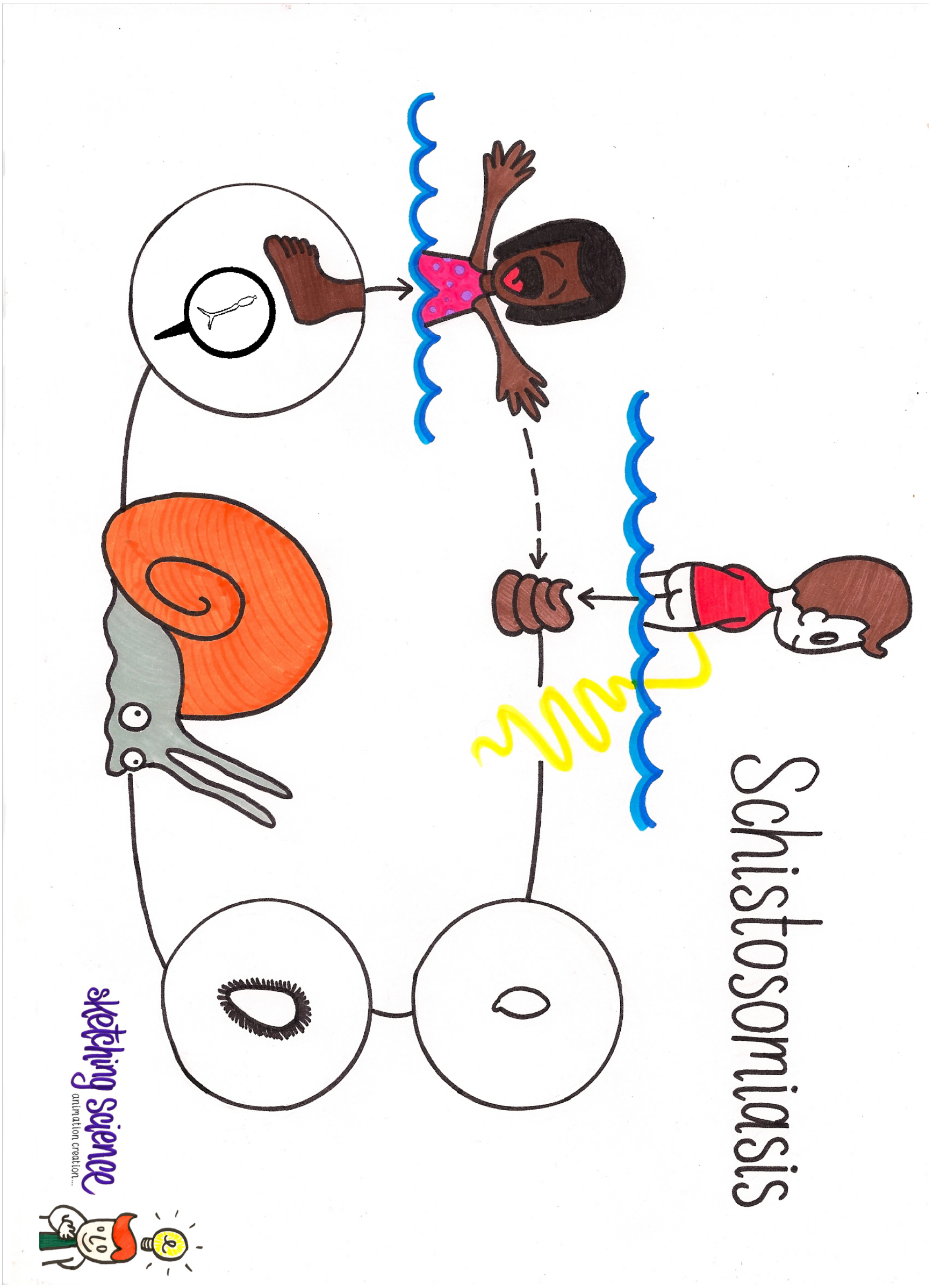

Fig S3. Educational resources used in Schistosome education: jigsaw puzzle. Image of the *Schistosoma* life cycle used as a poster and also cut into several laminated pieces to be used as a jigsaw puzzle.

BILARZIÔZY

Ao Nosivolo no misy ny bibin'aretina Bilarziôzy. Miditra amin'ny hoditra io bibin'aretina io rehefa mirobo-drano ny olona iray. Ary arakaraky ny hamaroan'ny bibin'aretina tafiditra anaty vatana no mamparary bebe kokoa. Izany hoe arakaraky ny maha-ela ny fotoana ilomana ao anaty Nosivolo no maha-maro ny bibin'aretina tafiditra.

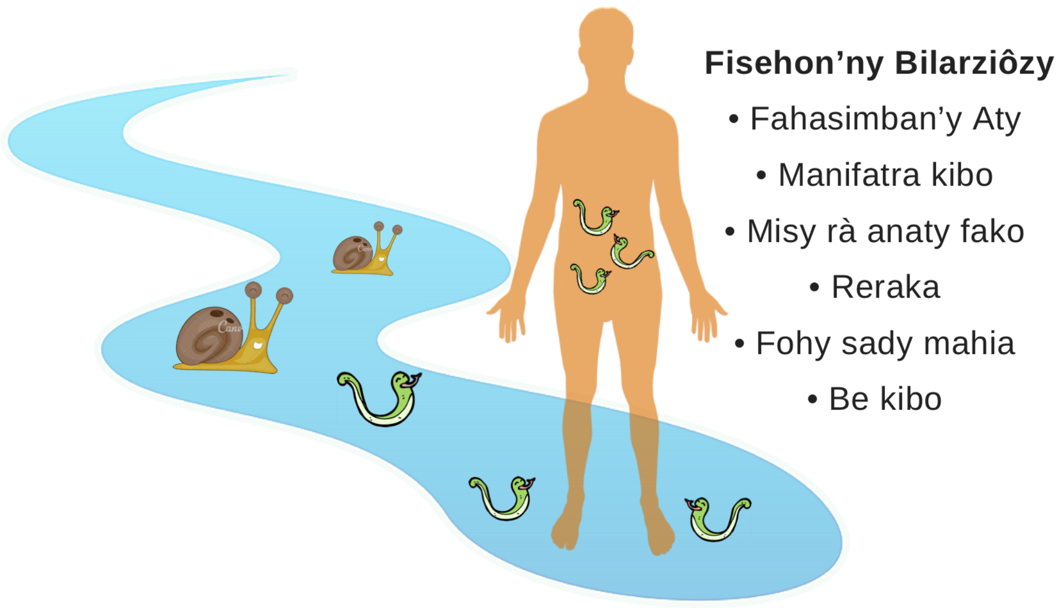

Fisehon'ny Bilarziôzy

- Fahasimban'y Aty
- Manifatra kibo
- Misy rà anaty fako
- Reraka
- Fohy sady mahia
- Be kibo

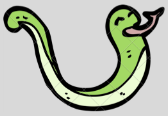

Ny fikaroahana natao dia nanambara fa ny akabeazan'ny ankizy aty amin'ny faritra misy antsika dia tratran'io aretina io daholo.

Fig S4. Educational resources used in Schistosome education: poster in Malagasy. Poster outlining important factual information about schistosomiasis, in Malagasy.

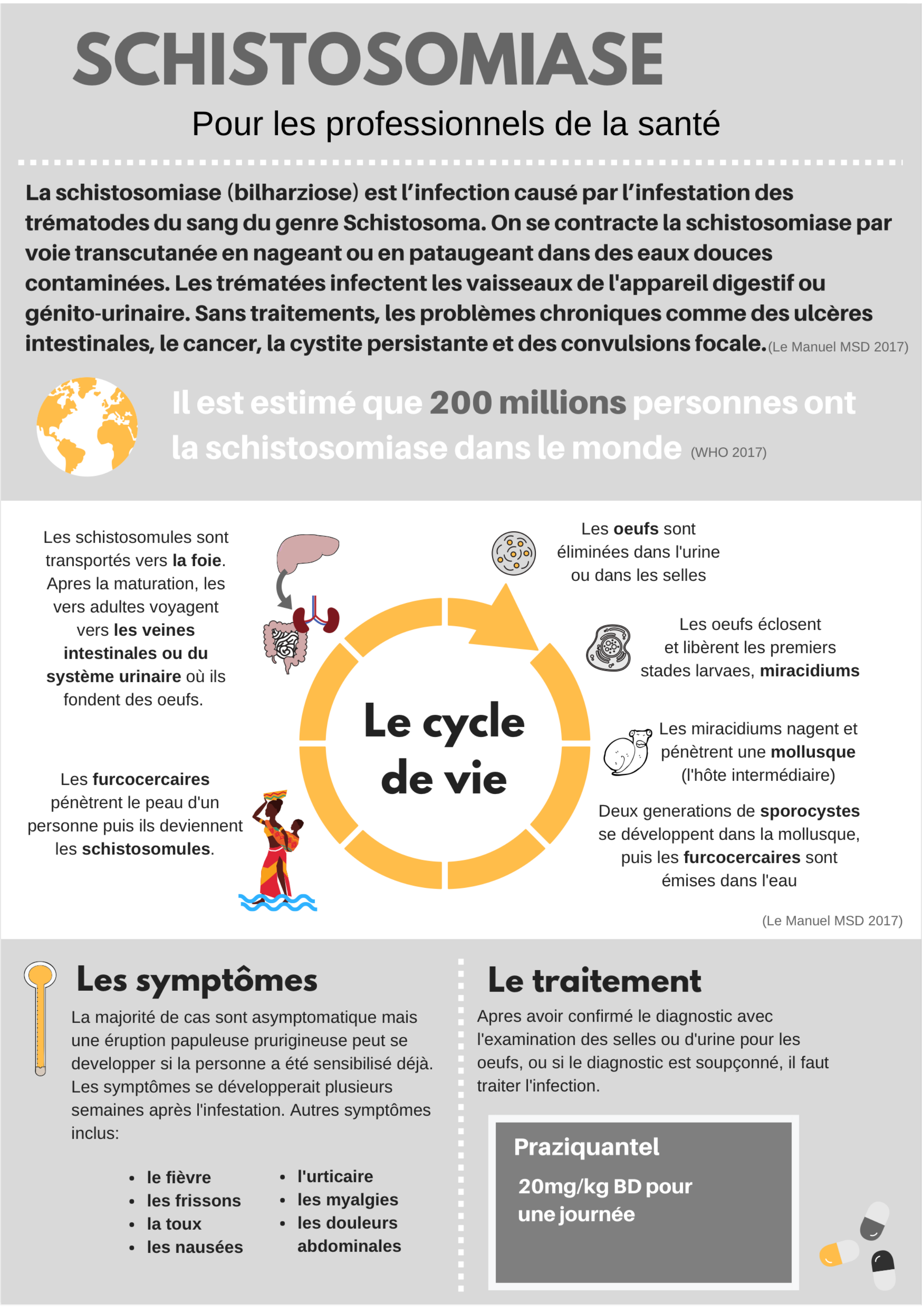

Fig S5. Educational resources used in Schistosome education: poster in French. Poster outlining important factual information about schistosomiasis, in French.

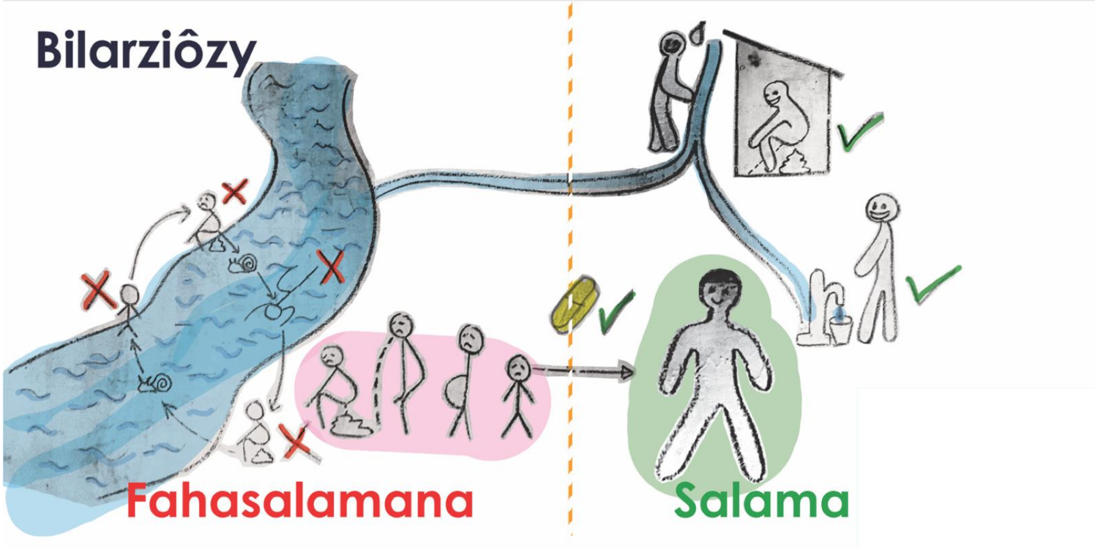

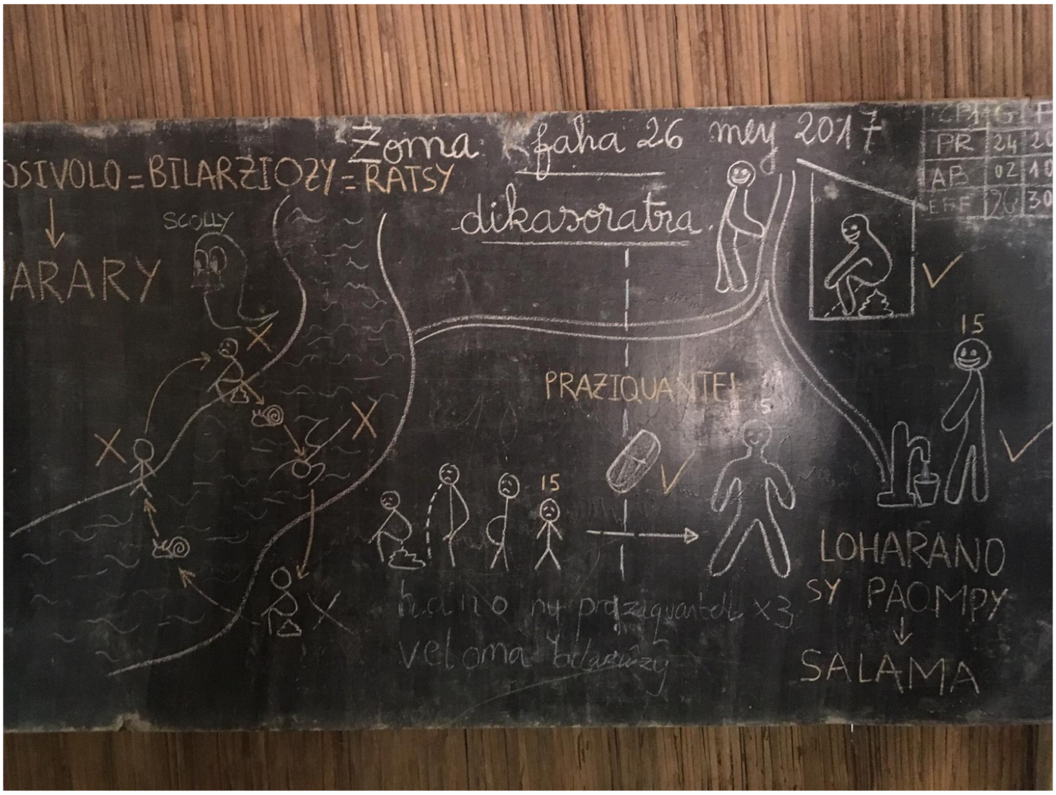

Fig S6. Diagrams used in classroom teaching. Top: Diagrams designed in collaboration between scientists, teachers and local community. Bottom: These diagrams which were also transcribed onto blackboards by local teachers in classrooms to guide relevant teaching on importance of schistosomiasis prevention, transmission and treatment.

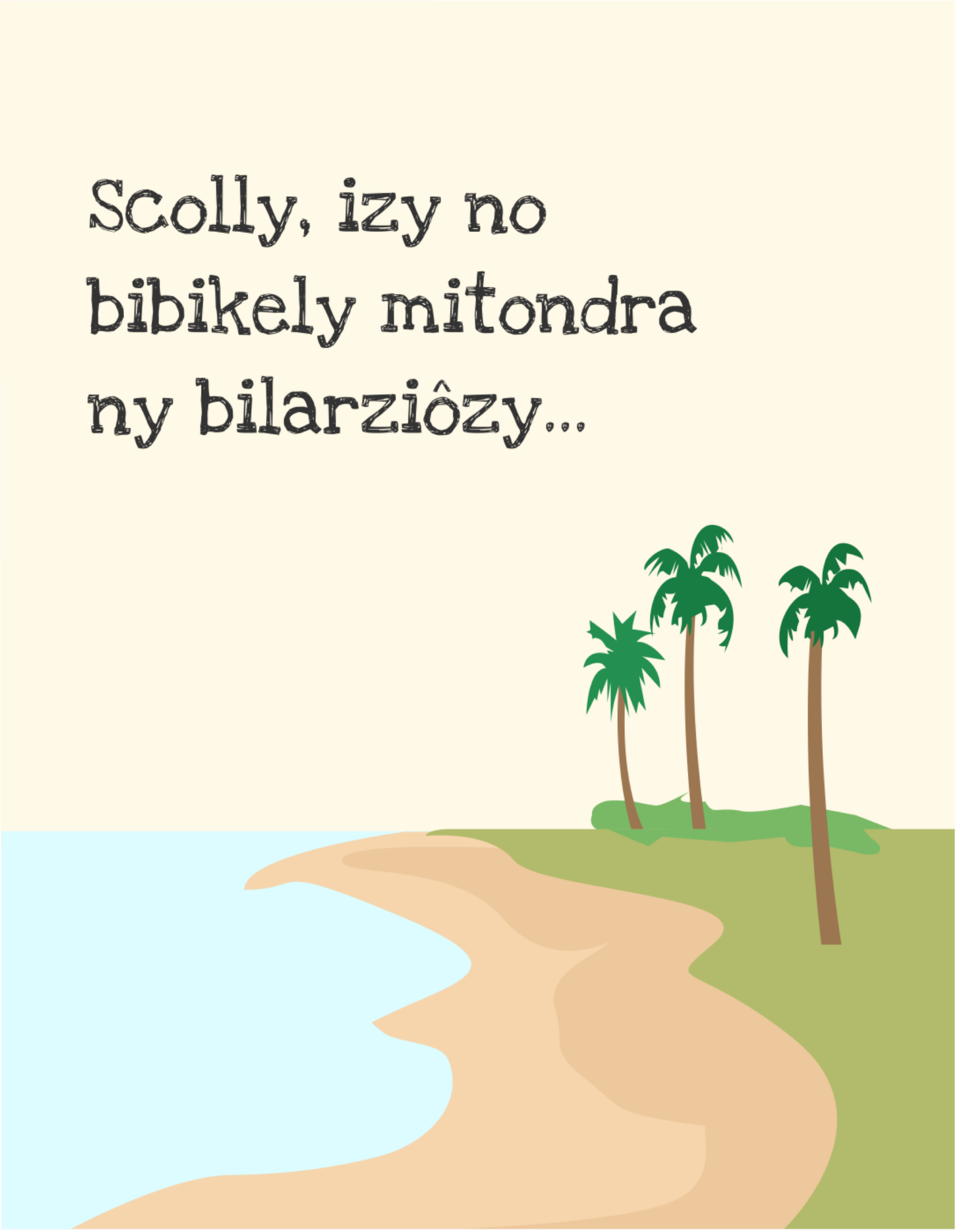

Fig S7. Cartoon book: "Scolly the Schisto Bug". This tells a story of how 'Scolly' the schistosome can infect people when they come into contact with the river, that children should defecate in latrines rather than the river to avoid passing Scolly back into the river, and that schistosomiasis can be treated with praziquantel. Written in Malagasy.

Tany alavi-davitra tany nisy rano nikoriana namakivaky ny Ala sy ny toeram-pambolena ary fiompiana. Tena manan-danja tokoa izy ity amin'ny olona mipetraka amin'ny faritra andalovany. Eo ny olona no midio, manasa ny lambany eny fa na dia ny trondro ataony sakafo ihany koa dia alainy ao. Toerana ipetrahan'ny zava-manan'aina maro ihany koa anefa izy ity, hatramin'ny trondro lehibe ka hatramin'ny kely, ao koa ny karazana kanakana kely.

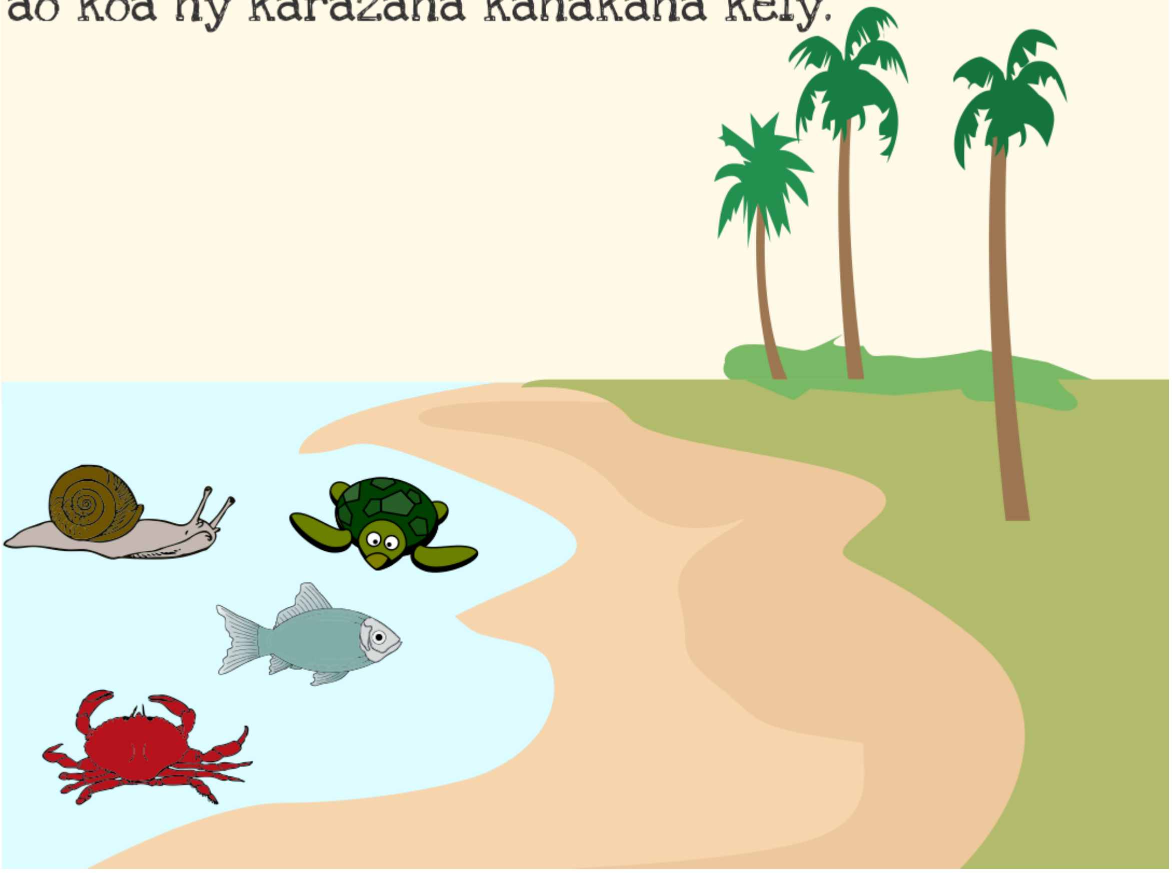

Fa ny tena zava-dehibe dia ao amin'io rano io no mipetraka i Scolly, izy no bibikely mitondra ny bilarziôzy

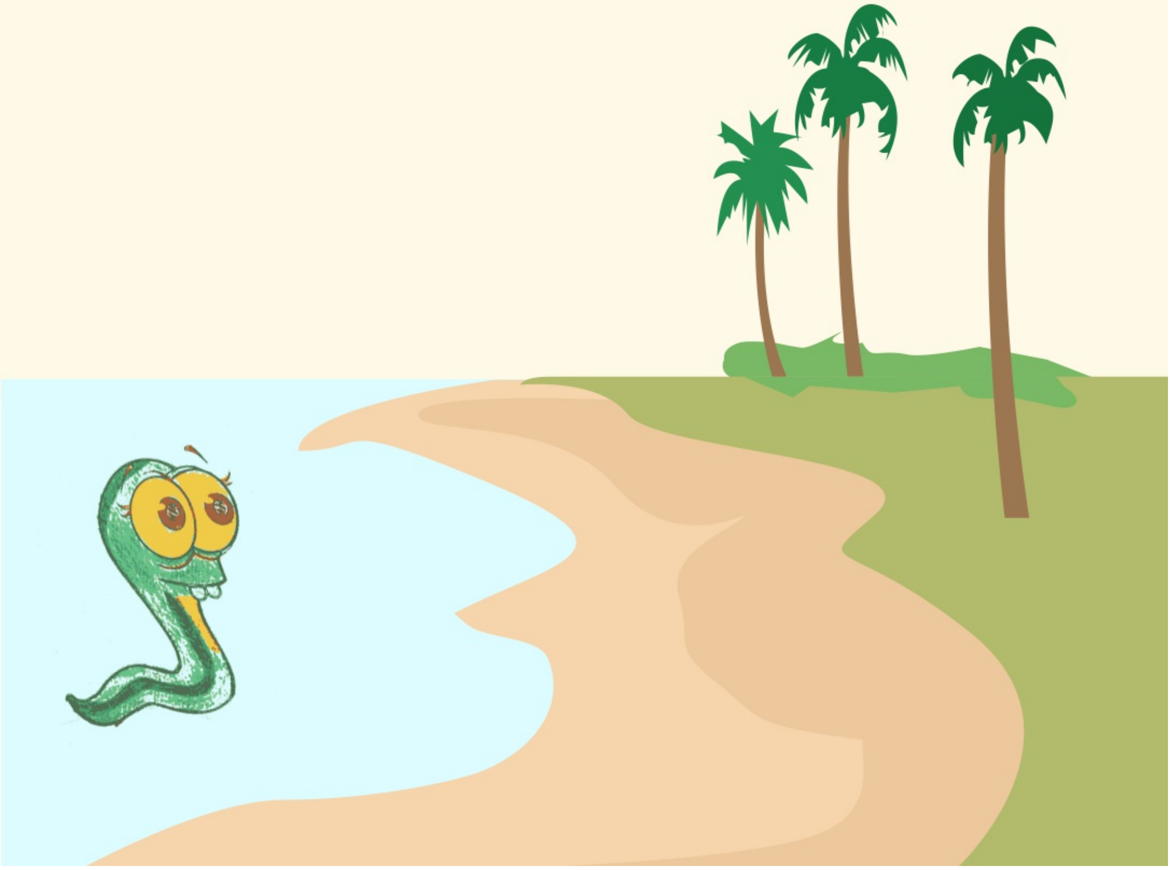

Scolly dia bibikely masika sy pelika. Mahafantatra azy daholo ny tsirairay noho izy mahatonga olana matetika. Rehefa andavan'andro i Scolly dia miara-miaina amin'i Sid Langato namany. Tsy afak'andro sy mora noana izy rehefa mipetraka an-trano ao amin'ny akoran'i Sid Langato izay maizina rahateo moa ka aleony mivoaka mitady zava-baovao ao anaty rano...

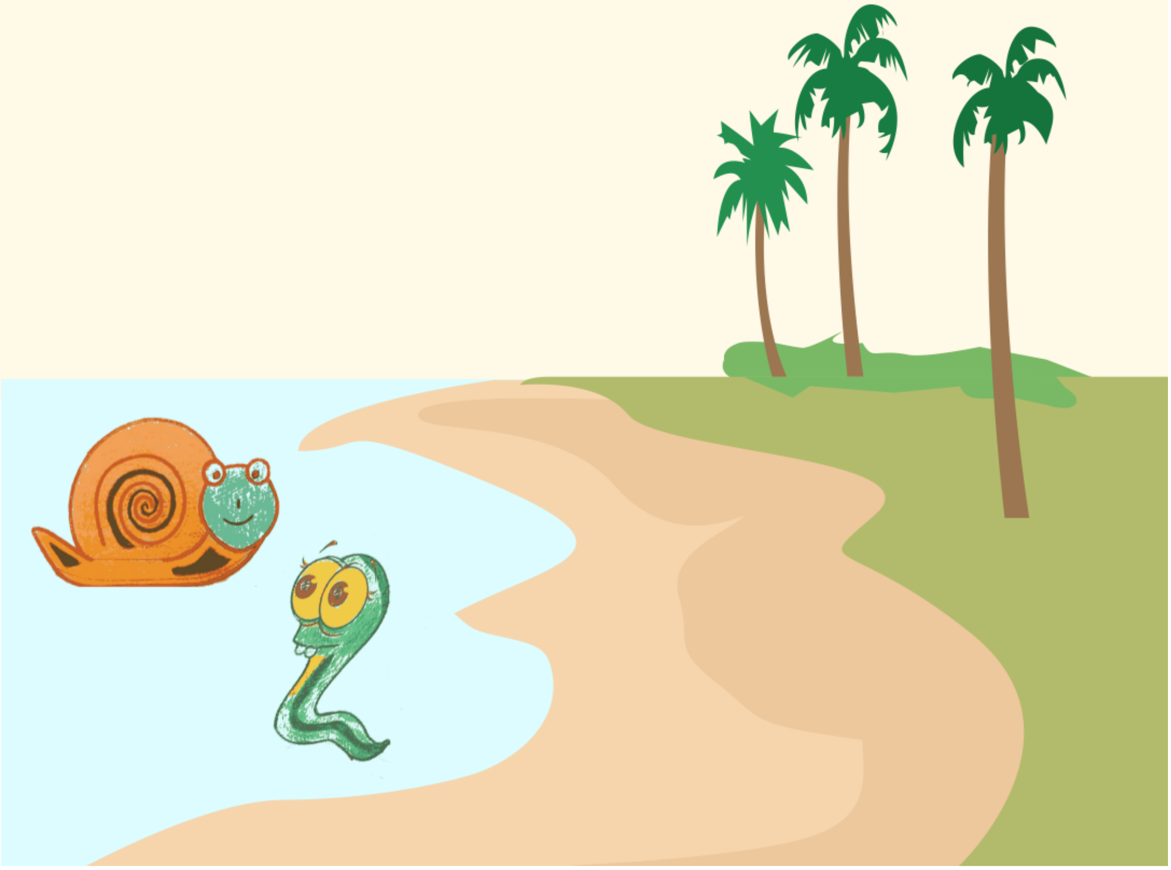

"Scolly a, mba hendry! Mandehana mody!" , izay no tenin'ny rehetra raha vantany vao mahita azy nefa hodiany tsy hainoina fa tohizany ihany izay tiany atao. Mba fantatrao ve oe inona no zavatra tiany?

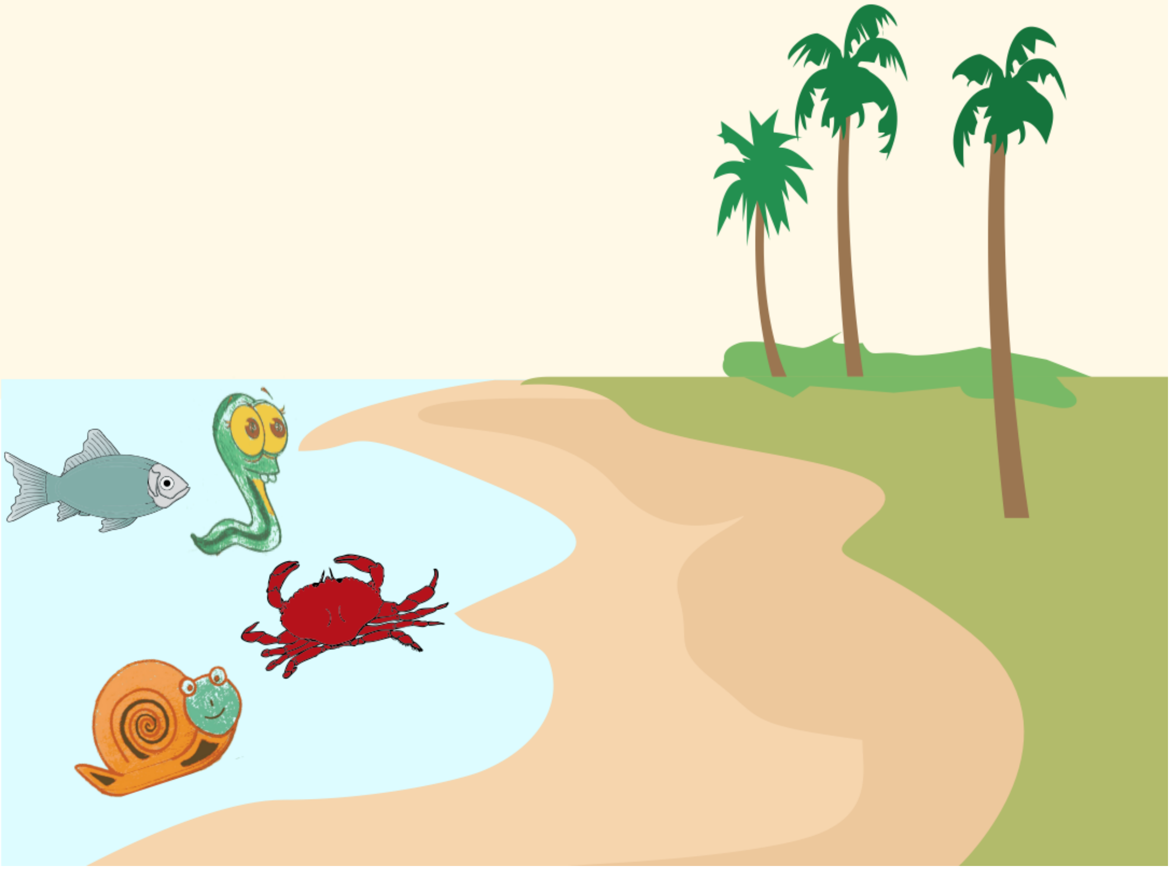

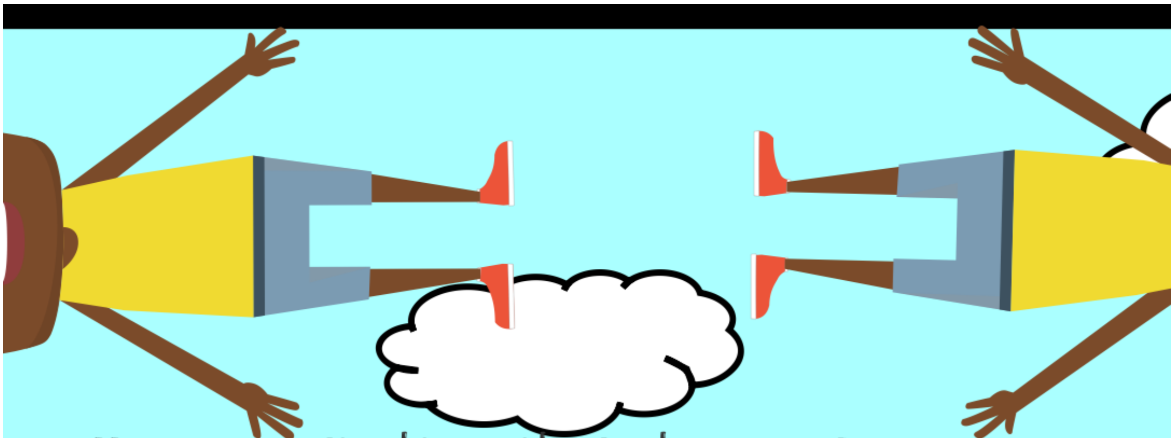

Scolly… Izy dia tia mitady toeram-baovao foana ipetrahany, tena tiany rehefa mafana mampahazo aina sy be-sakafo hohanina. Fantatrao ve oe mety ho aiza izao izany? Ambany vato ve? TSIA!... Anaty lavaka ambany rano ve? TSIA!... Anaty ranjon'ny olombelona ve? ENY dia eny tokoa! Mety mihevitra ianao hoe mba hafahafa koa izany nefa dia ao tokoa no tena tian'i Scolly hipetrahana.

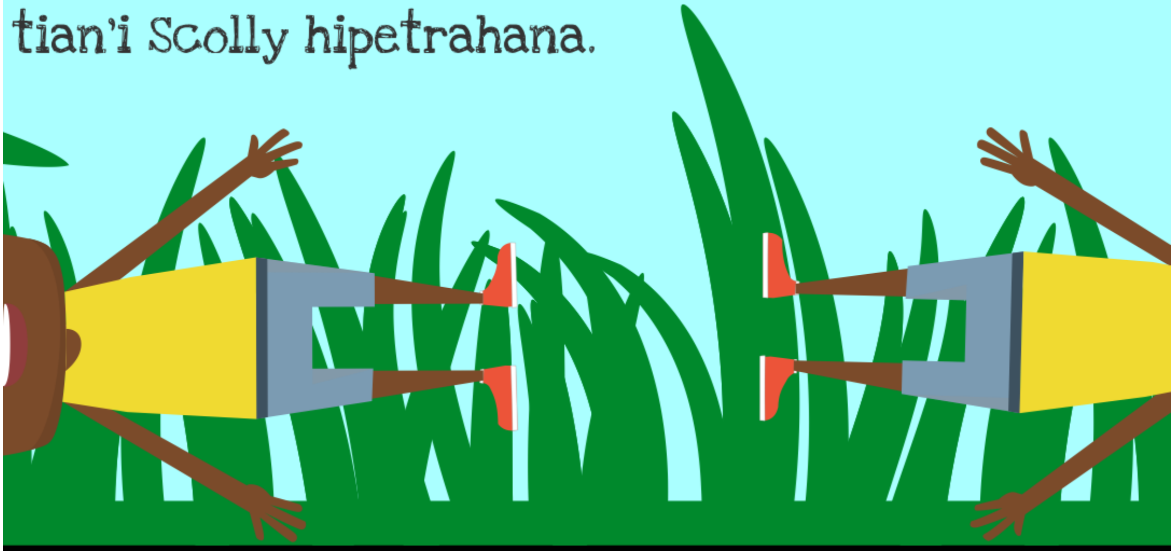

Mandany fotoana miaka-midina mitady tongotra itsofohana izy. Tsy mifidy izy na maloto na madio, na lava na fohy, na matavy na mahia fa tiany daholo! Ny hany hitany dia toerana mafana mahafinaritra sady feno rà azo iforetana tsara. Tsy manelingelina azy ny hoditra ivelany fa ilay rà matsiro ao anatiny no ilainy

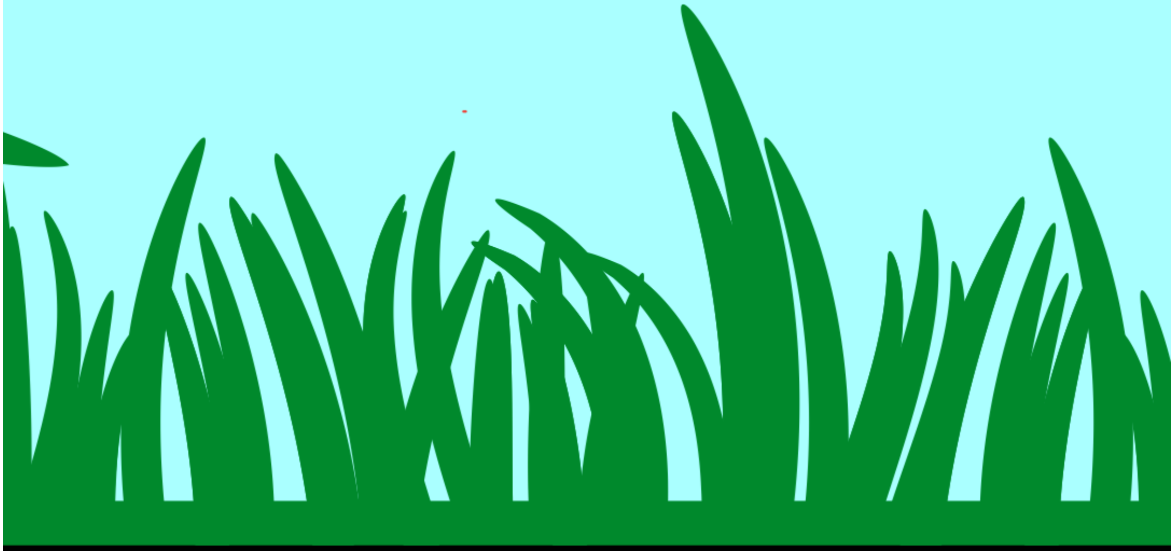

Izao dia hijery an'i Lullo isika. Manamorina ny rano no mipetraka ny olona eo an-tanàna, anisan'izany koa i Lullo sy ny fianakaviany. Zazalahy faly lava izy ary ny zavatra tiany indrindra dia ny milalao sy milomano eo amin'ny rano miaraka amin'ny namany.

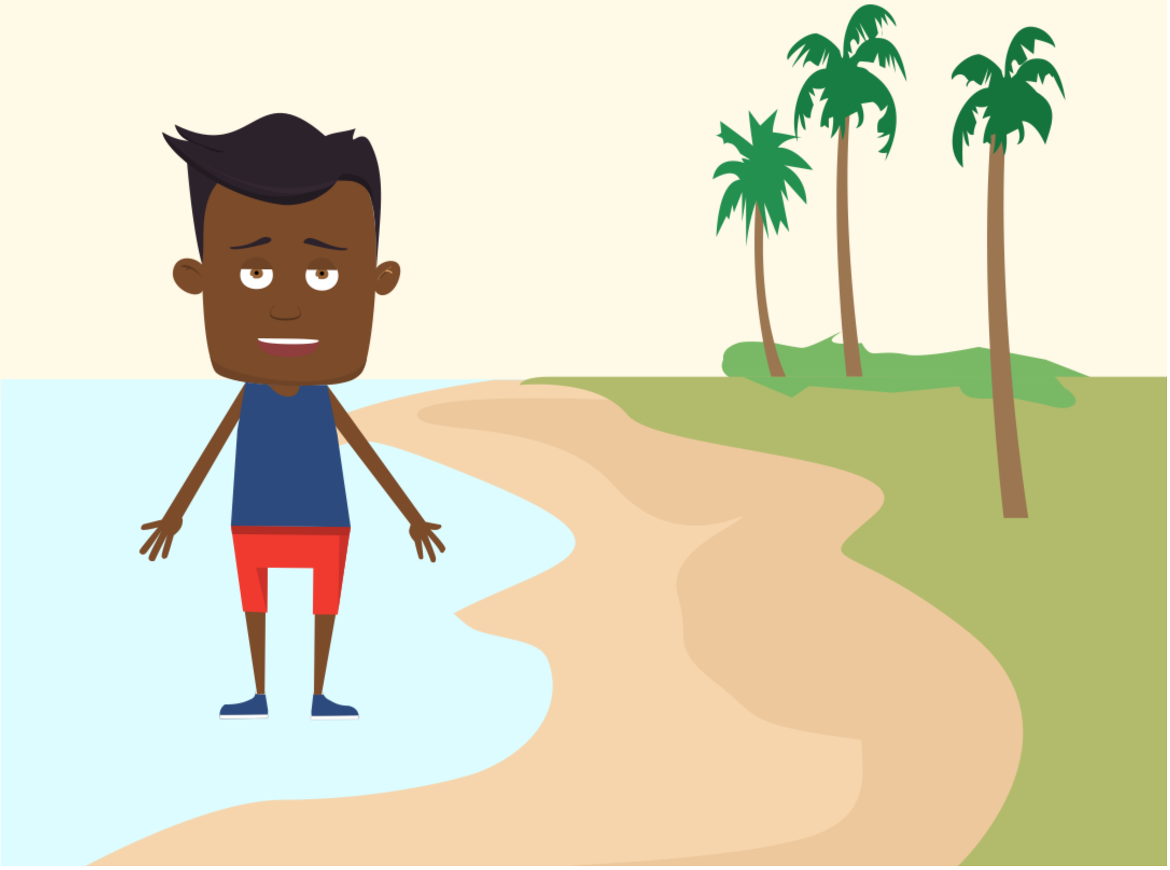

Kely sisa dia tratran'i Scolly i Lullo. Voamarik'i Scolly tao anaty rano ireo kitrokely mahafatifatin'i Lullo ary nanapa-kevitra izy fa ao no hipetraka manaraka. Tamin'i Lullo nipetraka tao anaty rano naka trondro ataony sakafo iny mihitsy no natahafidiran'i Scolly teo amin'ny lohaliny. Uh Oh!

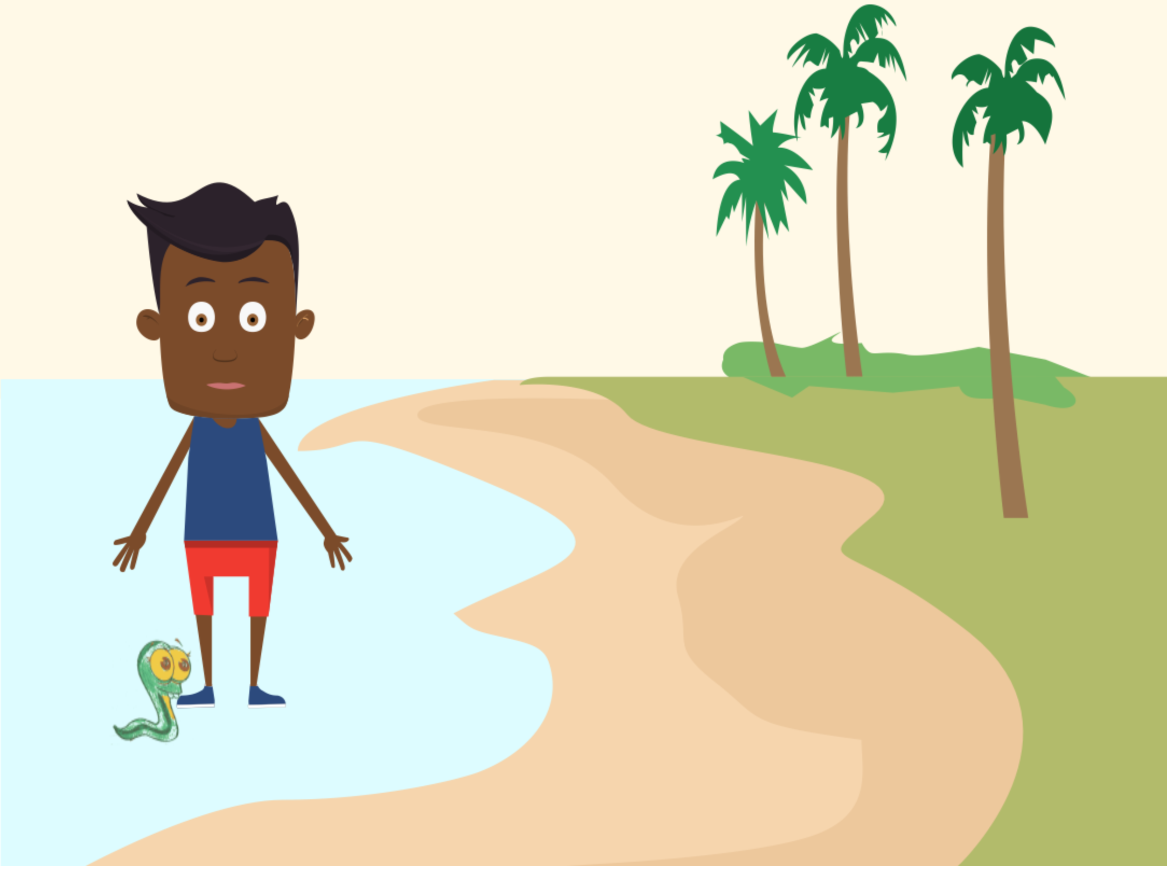

Ny ampitso maraina dia tsy salama mihitsy Lullo nifoha. Narary be izy sady nangery matetika. Dia nandeha nanao ny malotony tao anaty rano izy… Nahazo raharaha i Scolly manoloana ity rà matsiro-be an'i Lullo ka dia nanara-po mihitsy izy. Tsy vitan'ny nampaharary fotsiny an'i Lullo izy fa mbola nanapa-kevitra ny hiteraka! Lasa feno Scolly kely maro be koa i Lullo! Rehefa mangery ao anaty rano anefa i Lullo dia lasa milatsaka manaraka any koa ny sasany amin'ireo Scolly kely ka izay tonga anaty rano dia lasa mamonjy an'izay langato akaiky indrindra hitany…

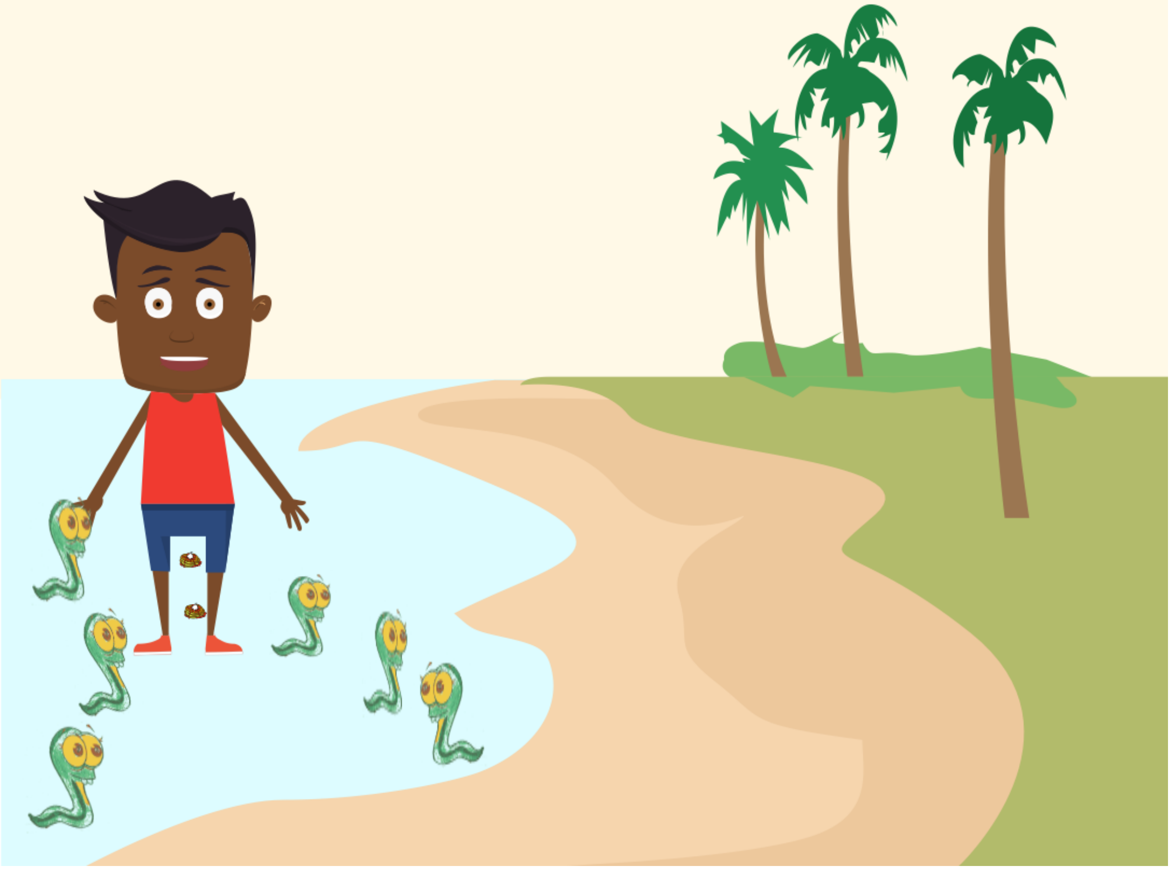

Ny andro manaraka an'io dia nandeha nisadodo tao anaty rano ry Mahir sy Miley naman'i Lullo. Efa noana sy laony amin'izay ireo Scolly kely tao anaty rano ka vantany vao nahita an'i Mahir sy Miley nilomano manodidina teo izy ireo dia tonga dia niditra aingana tao anaty vatan-dry Mahir hitady sakafo.

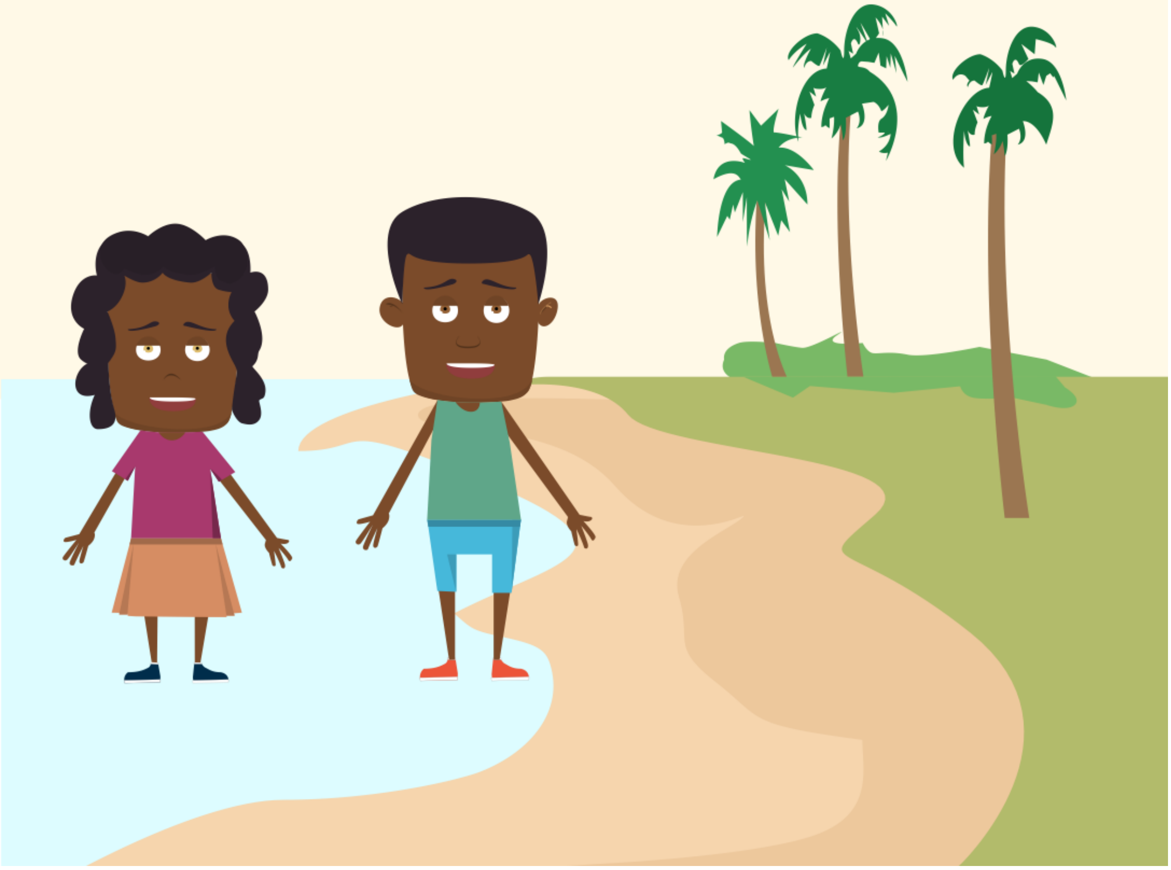

Tsy nahagaga raha tonga dia tsy salama koa Mahir sy Miley ny ampitso. Narary be ry zareo sady te hangery foana. Ireo izay rehetra nandeha an-drano nilalao na ninasa na nanjono dia lasa narary avokoa ary ny sasany aza dia tsy maintsy namonjy hopitaly. Izany rehetra izany noho ny filalaovana rano nisy an'i Scolly sy ny zanany. Nampalahelo Miley sy Mahir.

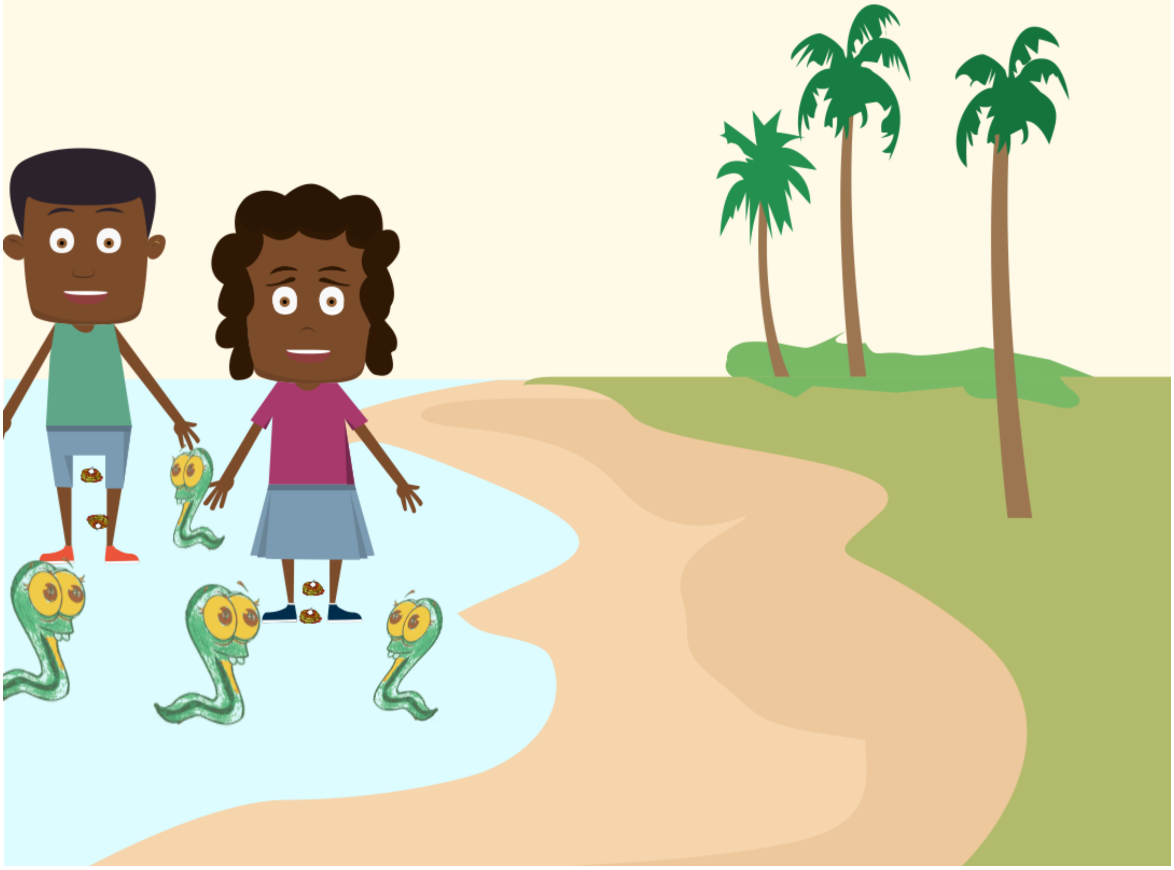

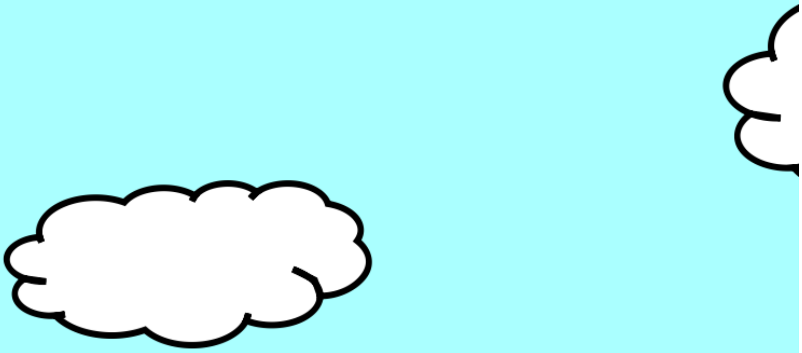

Ny Dokotera koa mahafantatra fa raha mampiasa kabone sy toeram-pidiovana ny ankizy ao an-tanàna ,fa tsy manao maloto an-drano ary mba milalao an-tanety fa tsy anaty rano dia nijanona tao anaty langato I Scolly fa tsy nifindra niteraka aretina ho an'ny olona…

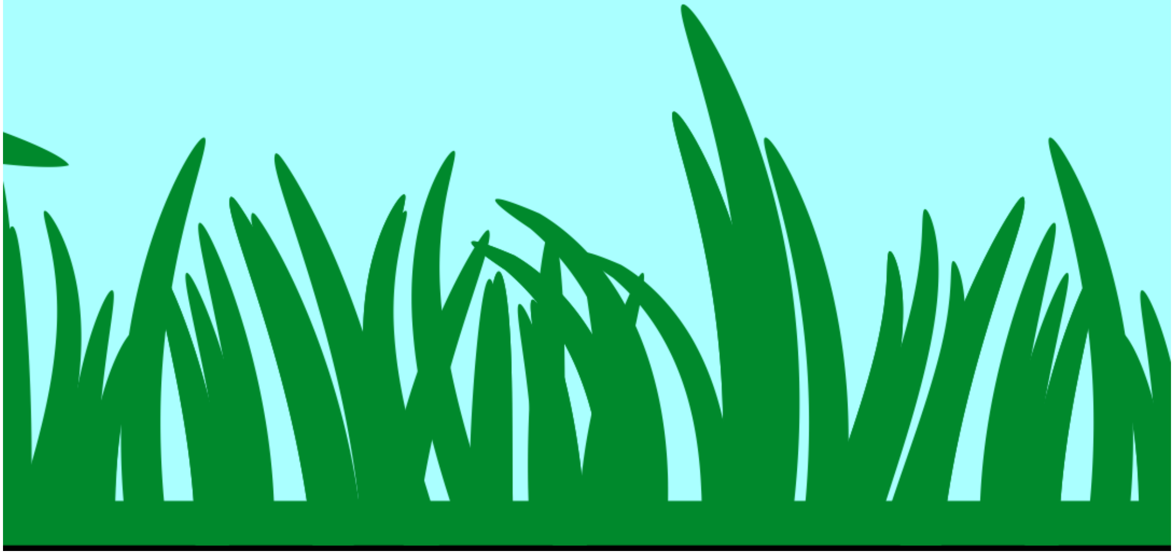

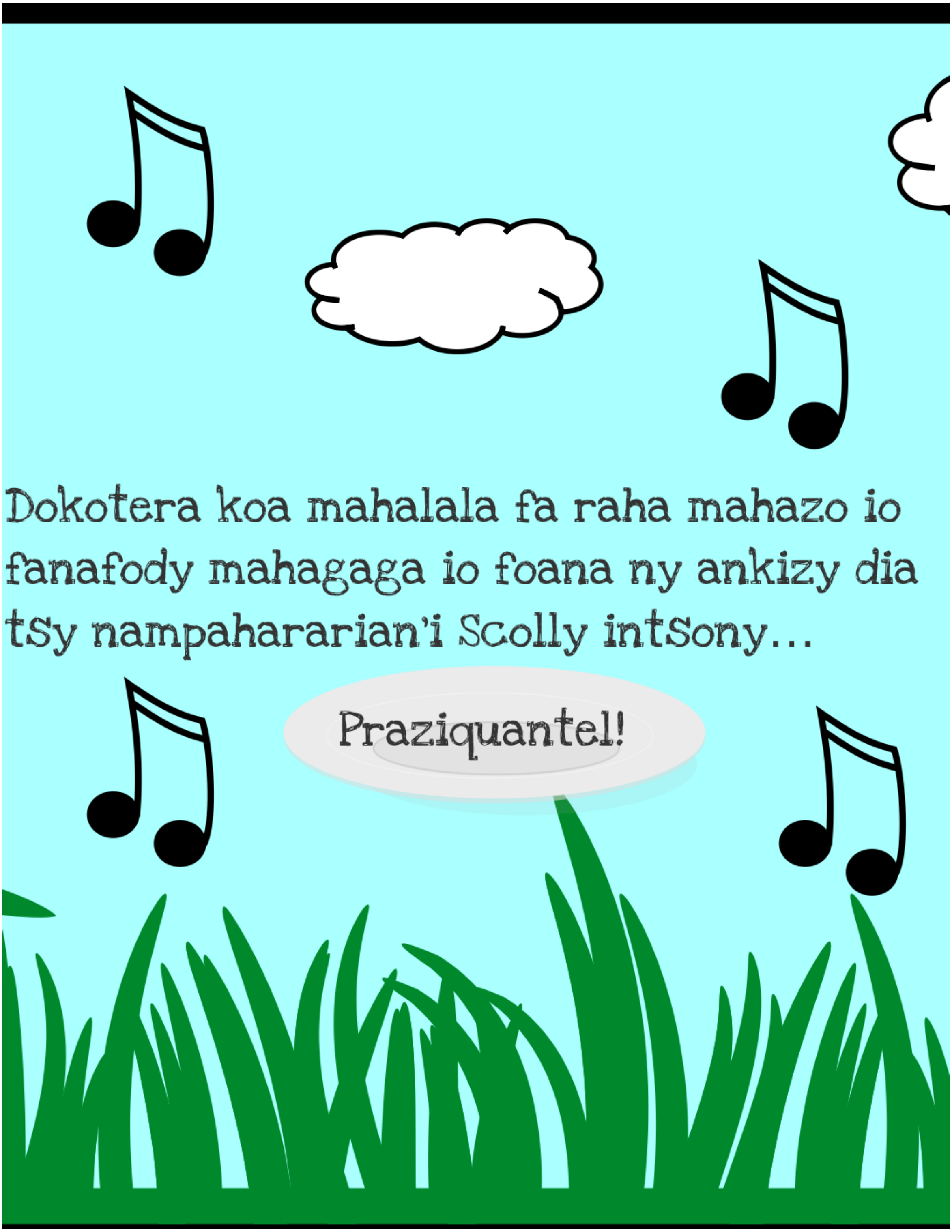
Dokotera koa mahalala fa raha mahazo io fanafody mahagaga io foana ny ankizy dia tsy nampahararian'i Scolly intsony…
Praziquantel!

Niverina nafana niaraka tamin'ny namany Sid langato i Scolly ary ny ankizy milalao salama sy finaritra…

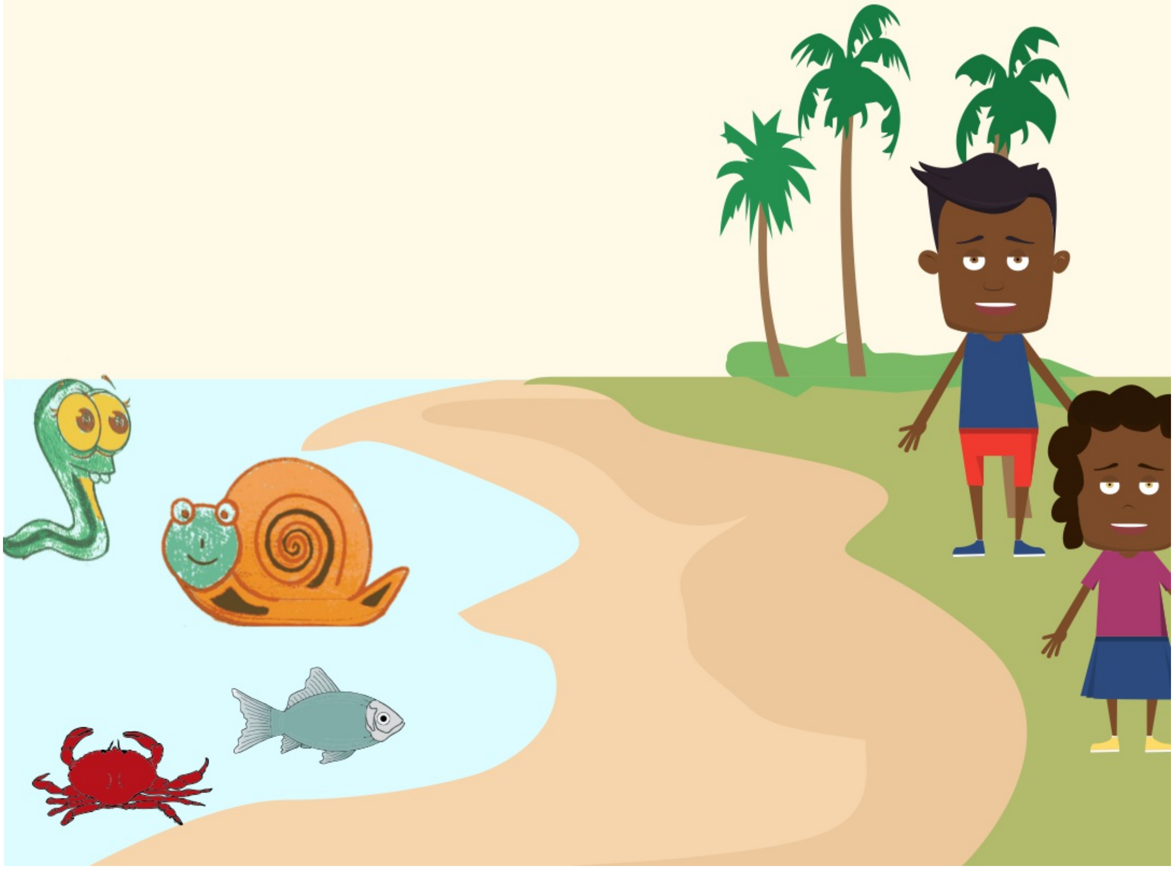

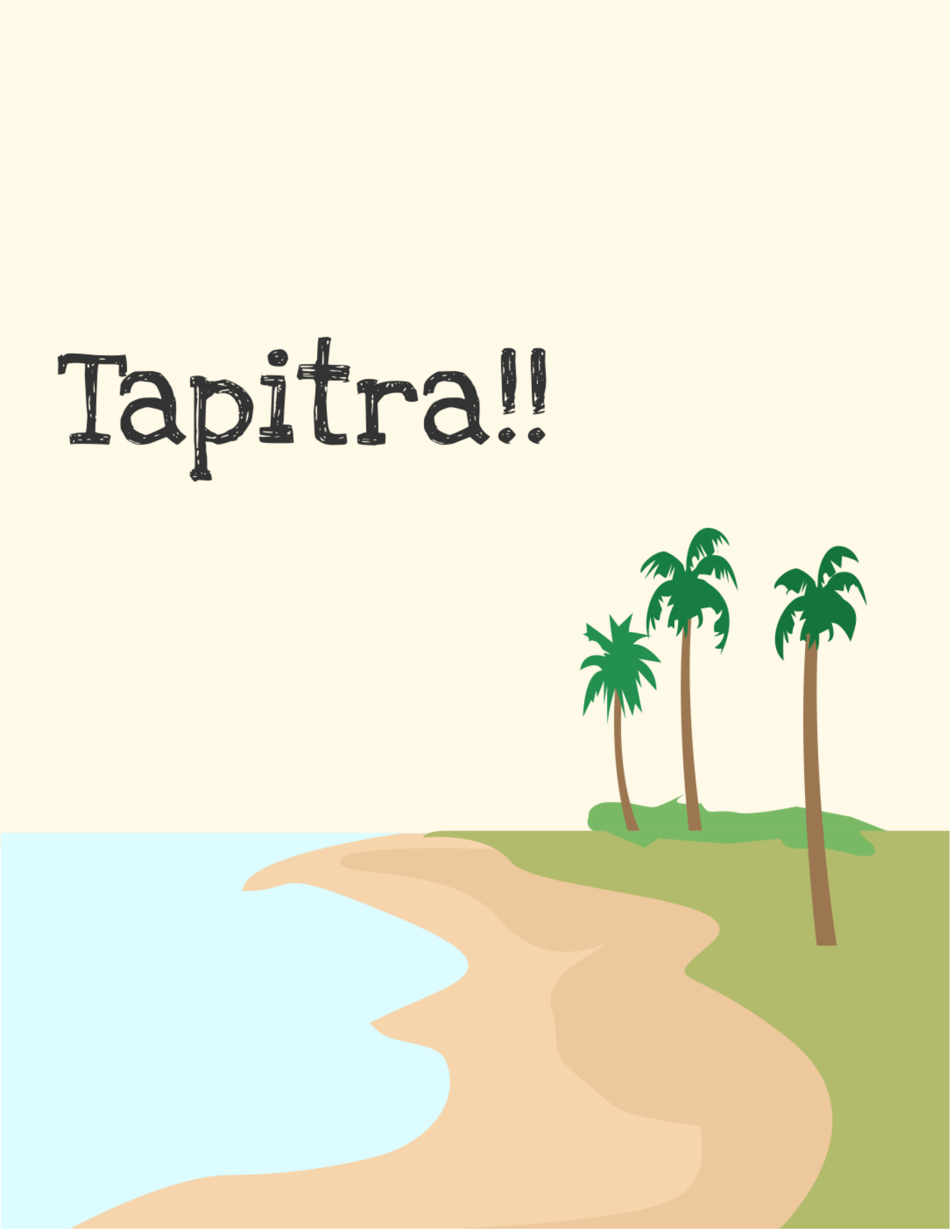
Tapitra!!

MADEX

INDIVIDUAL QUESTIONNAIRE

Schistosomiasis: education, treatment and research. A programme of implementation research to inform the effective and sustainable scaling-up of integrated neglected tropical disease control initiatives in Marolambo region of Madagascar.

Date: (dd/mm/yy): __ __ / __ __ / __ __ Interviewer Initials _______________________________________

Consent checklist

Has written consent been obtained? Y ☐ N ☐ *Only proceed if Yes*

A. Participant details

1. Participant ID: __ __ __ / __ __ __ / __ __ 2. School name: ___________________________

3. Participant's name: __________________________ 4: Gender: Male ☐ Female ☐

5. Is full date of birth known? Y ☐ N ☐

6. Date of birth (dd/mm/yy): __ __ / __ __ / __ __ 7: Current age: ________

KNOWLEDGE

Q 1: Have you heard of schistosomiasis/bilharzia? Y ☐ N ☐

Q2: In your own words, what is schistosomiasis/bilharzia?

....

....

Q3: How do you think you get infected with Schistosomiasis/bilharzia? *Tick all mentioned*

Playing with soil	
Swimming/bathing in infested water	
Dirty hands	
Eating contaminated food	
Drinking untreated water	
Snail	
Mosquito	
Germ/ virus	
Pollution	
Stress	
Don't know	
Other. Please state..	

File S1. KAP Questionnaire.

Q4: How do you feel when you get Schistosomiasis/bilharzia? What happens to you? *Tick all mentioned*

Diarrhoea	
Fever	
Blood in stool	
Vomiting	
Itching	
Tired	
Loss of appetite	
Cough	
Swollen abdomen	
Blood in urine	
Don't know	
Other. Please state.	

Q5: How long do you feel poorly for if you get schistosomiais/bilharzia?

For one day	
For one week	
For a few weeks	
For months	
For years	
Forever	
Until I get treated	
Until I go for a poo	
Don't know	
Other. Please state.	
Refused	

Q6: Can you prevent yourself from getting schistosomiasis/bilharzia? Y ☐ N ☐

Q7: If yes, how do you prevent schistosomiasis/bilharzia? *Tick all mentioned*

Avoid playing with soil	
Washing hands before eating	
Avoid swimming/bathing in ponds/streams	
Washing vegetables/fruit before eating	
Taking praziquantel or equivalent.	
Avoid drinking untreated water	
Avoid washing clothes in ponds/streams	
Avoid playing in infested water	
Don't know	

Other. Please state.	
Refused	

ATTITUDES

Q8: Do you worry about getting schistosomiasis/bilharzia? Y ☐ N ☐

Q9: Do you think schistosomiasis/ bilharzia is a serious disease? Y ☐ N ☐

Q10: Do you think there is any treatment for bilharzia/schistosomiasis? Y ☐ N ☐

Q11: If yes, what do you think the treatment is? ....

PRACTICES

Q12: Where do you go to the toilet to defecate? *Tick all mentioned*

Household toilet	
Village toilet	
School toilet	
Neighbour toilet	
On the ground	
In the river	
In the lake	
Don't know	
Other. Please specify.....	
Refused	

Q13: Where do you go to urinate? *Tick all mentioned*

Household toilet	
Village toilet	
School toilet	
Neighbour toilet	
On the ground	
In the river	
In the lake	
Don't know	
Other. Please specify	
Refused	

Q14: Does your house have a toilet? Y ☐ N ☐

Q15: If yes, do you use it? Y ☐ N ☐

Q16: If no to Q 11 what stops you? *Tick all mentioned*

Females not permitted when menstruating OR not suitable for both genders	
Toilet is dirty	
Toilet is broken	
Toilet pit is full/overflowing	
Toilet is not suitable for children	
Toilet is in an unsafe location	
Must pay a fee to use it	
Toilet is too far away	
Don't know	
Other. Please specify	
Refused	

Q17: Do you cross the river to school? Y ☐ N ☐

Q18: How often do you wash yourself in the river?

Every day in the morning	
Every day in the afternoon	
Every day morning and afternoon	
1-2 times per week	
1-2 times per fortnight	
1-2 times per month	
Other. Please specify.	

Q19: How often do you swim or play in the river?

Every day in the morning	
Every day in the afternoon	
Every day morning and afternoon	
1-2 times per week	
1-2 times per fortnight	
1-2 times per month	
Other. Please specify.	

Q20: How often do you fetch water for drinking?

Every day in the morning	
Every day in the afternoon	
Every day morning and afternoon	
1-2 times per week	
1-2 times per fortnight	
1-2 times per month	
Other. Please specify.	

Q21: How often do you wash clothes in the river?

Every day in the morning	
Every day in the afternoon	
Every day morning and afternoon	
1-2 times per week	
1-2 times per fortnight	
1-2 times per month	
Other. Please specify.	

Q22: How often do you wash plates in the river?

Every day in the morning	
Every day in the afternoon	
Every day morning and afternoon	
1-2 times per week	
1-2 times per fortnight	
1-2 times per month	
Other. Please specify.	

Q23: How often do you wear shoes when you go outside?

	Always	Sometimes	Never
When inside your home			
When outside your home			
When defecating/urinating			

Q24:

A) To your knowledge, have you taken albendazole in the last 12 months? Y ☐ N ☐

B) To your knowledge, have you taken praziquantel in the last 12 months? Y ☐ N ☐

Thank you very much for completing the questionnaire!
